# Repurposing of lansoprazole and nitazoxanide as NR2F2 inhibitors in the gastric cancer cell line GCIY: computational insights and siRNA-mediated *in vitro* tests

**DOI:** 10.3389/fchem.2026.1916222

**Published:** 2026-08-25

**Authors:** Giulia Culletta, Muhammad Sohaib Roomi, Maria Azzurra Caricasulo, Adriana Zanetti, Mineko Terao, Gabriela Paroni, Enrico Garattini, Marco Tutone

**Affiliations:** 1 Department of Biological, Chemical and Pharmaceutical Sciences and Technologies (STEBICEF), University of Palermo, Palermo, Italy; 2 Department of Biochemistry and Molecular Pharmacology, Istituto di Ricerche Farmacologiche Mario Negri IRCCS, Milan, Italy

**Keywords:** binding pose metadynamics, blind docking, COUP-TFII, gastric cancer cell line GCIY, lansoprazole, molecular dynamics, nitazoxanide, NR2F2 nuclear receptor

## Abstract

**Background:**

Nuclear receptors (NRs) are a superfamily of ligand-activated transcription factors that mediate the cellular response to hormones, vitamins, and dietary lipids, thus orchestrating numerous physiological processes and influencing multiple disease states. Within this superfamily, NR2Fs, also known as Chicken Ovalbumin Upstream Promoter Transcription Factor (COUP-TF), are a family of nuclear orphan receptors, due to the lack of known endogenous ligands. Among them, NR2F2 transcription factor activities involve regulation of cell differentiation during organogenesis, maintenance of adult tissue homeostasis, and tumorigenesis. To date, only the CIA compounds have been identified as inhibitors of NR2F2 driven transcriptional activity in prostate cancer cell lines, with the IC50 of CIA1 falling in the lower μM range.

**Methods:**

A comprehensive computational analysis has been conducted in order to gain insight into the inhibitory activity of the prototype CIA1 against NR2F2 at a molecular level. We leveraged the outputs of computational analysis to perform a virtual screening campaign to FDA compounds capable of binding to NR2F2. Luciferase reporter assay was used to functionally validate the interaction. RNA interference approaches were used to validate the specificity of NR2F2 activity on cell proliferation.

**Results:**

We identified lapatinib, glyburide, nitazoxanide, and lansoprazole as modulators of NR2F2-dependent reporter activity. In the gastric cancer cell line GCIY, lansoprazole and nitazoxanide exert antiproliferative effects that are mitigated by NR2F2 silencing. The convergence between *in silico* predictions and LBD-dependent reporter modulation supports the interpretation that lapatinib, glyburide, nitazoxanide, and lansoprazole represent *bona fide* functional interactors of NR2F2.

**Conclusion:**

Taken together, our data extend current evidence that NR2F2 is a ligandable orphan receptor whose transcriptional output can be pharmacologically tuned. Moreover, our findings provide new chemical tools for probing NR2F2 biology and nominate clinically used molecules as starting points for therapeutic or repurposing strategies targeting NR2F2-dependent programs.

## 1 Introduction

**FIGURE 1:**
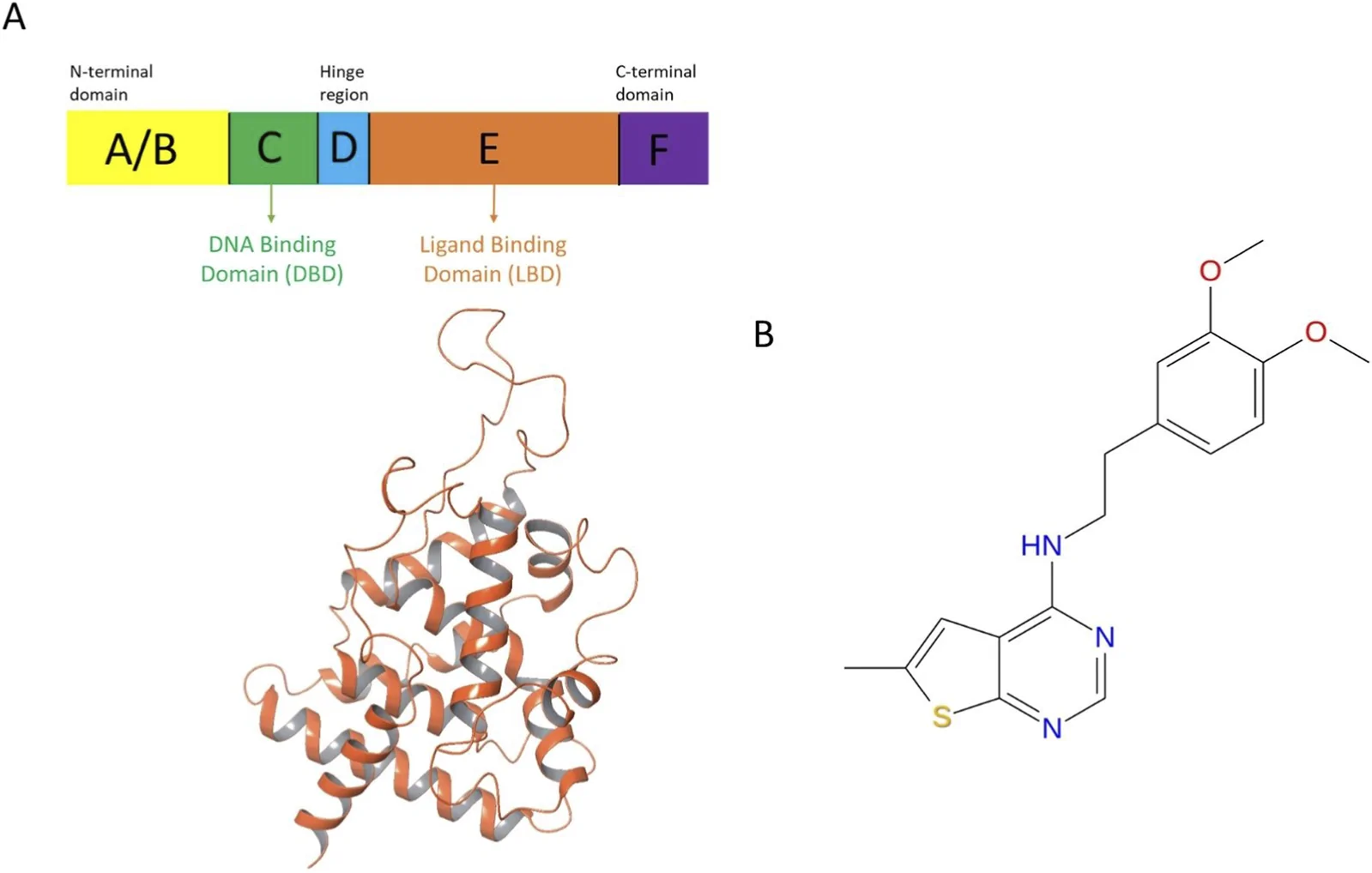
The x-ray 3D structure of the human COUP-TFII ligand binding domain (PDB ID: 3CJW) **(A)** and CIA1 compound **(B)**.

Nuclear hormone receptors (NRs) are an ancient superfamily of 48 transcription human factors (TFs) (Laudet, 1997). NRs functions are often dictated by interactions with specific ligands, including steroids, thyroid hormones, and retinoids. Once in the nucleus, NR acts as transcriptional regulators with direct DNA binding activity, thereby modulating the expression of target genes (Coppola and Waxman, 2021). Some NRs are classified as orphan receptors because there is no knowledge about endogenous ligands (Burris et al., 2013; Germain et al., 2006). Among these, there are the Nuclear Receptor Subfamily 2 group F members (NR2Fs), originally named Chicken Ovalbumin Upstream Promoter Transcription Factors (COUP-TFs) due to their ability to bind to the regulatory DNA sequences upstream of the chicken ovalbumin gene promoter. This subfamily has three members: NR2F1 (aka COUP-TFI, EAR-3), NR2F2 (aka COUP-TFII, ARP-1), and NR2F6 (aka COUP-TFIII, EAR-2) (Sajinovic and Baier, 2023). NRs exhibit a distinctive modular structure composed of five to six homologous domains, designated A to F (from the N-terminal to the C-terminal end) (Germain et al., 2006). Two defining domains of NRs are well characterized: the DNA-binding domain (DBD; region C) and the ligand-binding domain (LBD; region E) (Figure 1A). These regions demonstrate high levels of conservation, with the capacity to function independently. The variable N-terminal regions A/B and D exhibit less conservation, while the F region at the C-terminus is absent in some receptors and its function remains unclear. Notwithstanding the considerable degree of homology observed between the DBDs and LBDs of NR2F1 and NR2F2, with similarity ratings of 98 percent and 96 percent, respectively, it is noteworthy that NR2F6 displays the greatest divergence in sequence. Nevertheless, its functional relationship with the other family members remains closely related (Alexander et al., 2019; Germain et al., 2006; Sajinovic and Baier, 2023).

NR2F1 is predominantly expressed in the developing peripheral and central nervous system and is regarded as one of the main transcriptional regulators of cortical development, cell-type specification, and maturation (Bertacchi et al., 2019). NR2F2 regulates many intracellular signaling pathways and is implicated in several physiological, pathological, and developmental processes (Sajinovic and Baier, 2023). It plays a key role in tissue homeostasis and maintenance, functioning as a major regulator of cell differentiation and angiogenesis (Chen et al., 2012; Pereira et al., 1999; Wu et al., 2015; Yamazaki et al., 2009). Some studies have elucidated the role of NR2F2 in a variety of metabolic systems, such as adipogenesis, lipid metabolism, insulin secretion, and hepatic gluconeogenesis (Ashraf et al., 2019; Boutant et al., 2012; Li et al., 2009; Pereira et al., 1999). Furthermore, NR2F2 is essential for both male and female fertility (Oh et al., 2023; Qin et al., 2008). In contrast, NR2F6 has been investigated in various immune cells and adipocyte differentiation (Hermann-Kleiter et al., 2015; Sajinovic and Baier, 2023).

In carcinogenesis, the functions of NR2F family members have been hypothesized to be either negative or positive in cancer progression, based on the cell type and biological processes studied.

Mauri et al. have identified NR2F2 as a key regulator of cancer stem cell functions in malignant skin squamous cell carcinoma. They have been demonstrated to promote tumor renewal and restrict differentiation, thereby sustaining a malignant tumor state (Mauri et al., 2021). Xia et al. hypothesized that insulin promotes breast cancer cell invasion and migration by increasing the expression of NR2F2, a critical factor in the process of insulin-mediated breast cancer cell invasion and migration through its effect on the epithelial-mesenchymal transition (Xia et al., 2020). Moreover, inhibition of NR2F2 restores hormone therapy response to endocrine refractory breast cancers (Cai et al., 2025).

Another significant oncogenic role of NR2F2 is in the promotion of prostate cancer development and metastasis. The regulation of transforming growth factor-β (TGF-β) signaling, the fork head box protein m1 (foxm1)/centromere protein f (cenpf) pathway, and cancer metabolism have been identified as the key factors in this process. The suppression of COUP-TFII expression has been demonstrated to impede prostate cancer progression and metastasis to a substantial degree, thus implicating the protein as a potential significant therapeutic target for prostate cancer (Wang et al., 2020).

Feng and colleagues showed that the binding of NR2F2 can suppress gastric cancer proliferation and metastasis (Feng et al., 2017). Due to its ability to regulate many intracellular signaling pathways, NR2F2 presents itself as an interesting pharmaceutical target.

To date, Wang et al. using a high-throughput screening approach have identified the only potent and specific NR2F2 inhibitor for prostate cancer treatment (Le Guével et al., 2017; Wang et al., 2020). The inhibitor, CIA1, a N-[2-(3,4-dimethoxyphenyl)ethyl]-6-methylthieno [2,3-d]pyrimidin-4-amine hydrochloride (Figure 1B), was observed to bind directly to the NR2F2 ligand binding domain, thereby disrupting the interaction with transcriptional regulators, including FOXA1. This resulted in the suppression of NR2F2 activity in target gene regulation. *In vitro*, CIA1 has been demonstrated to inhibit the growth of multiple prostate cancer cell lines, with the IC_50_ observed to range from 1.2 to 7.6 μM. Furthermore, the molecule has been shown to suppress NR2F2 activity, thereby regulating its target genes. *In vivo* studies have demonstrated that the molecule can reduce tumor growth in prostate cancer xenograft mouse models.

Considering the biological evidence presented above, the present study aims to evaluate the potential active sites of the ligand-binding domain of NR2F2 and highlight the molecular mechanism of interaction with the goal of identifying more potent inhibitors. Artificial intelligence (AI), molecular docking, molecular dynamics simulations, and structure-based virtual screening have revolutionized medicinal chemistry and drug development by accelerating the identification of drug–target interactions and improving the efficiency of drug discovery. AI enhances the prediction of promising drug candidates by analyzing large biological and chemical datasets, while AI-enhanced molecular docking improves the accuracy of predicting how small molecules bind to target proteins (Mullowney et al., 2023; Zhang et al., 2025. Molecular dynamics simulations provide detailed insights into the stability and behavior of protein–ligand complexes under realistic physiological conditions, enabling researchers to better understand binding mechanisms (Stavrogiannis et al., 2026, Almerico et al., 2012). Structure-based virtual screening allows thousands to millions of compounds to be rapidly evaluated against therapeutic targets, significantly reducing the time and cost of experimental screening (Huang et al., 2024; Xu et al., 2021; Pibiri et al., 2020; Almerico et al., 2012). These computational approaches also facilitate the repurposing of approved drugs by identifying new therapeutic targets and uncovering previously unrecognized biological activities, providing a faster and more cost-effective alternative to *de novo* drug development (Fawzy et al., 2026). Collectively, these technologies have transformed medicinal chemistry by enabling the faster development of safer and more effective therapeutic agents. To this end, a series of computational studies were conducted, with an increase in accuracy, to assess the druggability of potential active sites using the compound CIA1 as a reference compound. The process started with the identification of potential binding sites and continued with docking, Binding-Pose-Metadynamics (BPMD) and unbiased Molecular Dynamics (MD). Finally, we employed MM-GBSA (Molecular Mechanics-Generalized Born Surface Area Continuum Solvation) free energy calculations. We used these outputs to perform a virtual screening of the DrugBank and FDA libraries. We tested the most promising hit compounds to assess their ability to modulate NR2F2-dependent transcriptional activity. Finally, we assessed their effects on gastric cancer cell proliferation and investigated the specificity of NR2F2-mediated activity.

## 2 Materials and methods

### 2.1 System and ligand preparation

The crystal structure of the human COUP-TFII ligand binding domain (PDB ID: 3CJW) was used. The structure was optimized using the Protein Preparation Wizard in Maestro (Schrödinger, New York, 2024-1) adding bond orders and hydrogen atoms to the crystal structure using the OPLS4 force field (Lu et al., 2021). Prime (Schrödinger New York, 2024-1) was used to fix missing loops, adding Pro268-Val286 residues to the protein and removing co-crystallized water molecules. The protonation state of ionizable protein groups was fixed at pH 7.2 ± 0.2 by using PROPKA. The polar groups such as hydroxyls, thiols, and amides were adjusted to optimize hydrogen bonds. The entire system was minimized considering an RMSD of 0.3 Å. Compound CIA1 was prepared using LigPrep (Schrödinger New York, 2024-1) with the force field OPLS4. Epik 3.9 (Schrödinger New York, 2024-1) was selected as an ionization tool at pH 7.2 ± 0.2 for CIA1. No tautomers were generated and a maximum number of conformers of 32.

### 2.2 SiteMap and blind docking

Potential ligand-binding pockets within the NR2F2 ligand-binding domain were identified using SiteMap (Schrödinger Release 2024-1, New York, United States) with standard parameter settings (Halgren, 2007; Halgren, 2009). SiteMap applies a grid-based algorithm to evaluate the protein surface and detect regions that may accommodate small-molecule ligands, including previously uncharacterized or allosteric cavities. The procedure generates clusters of favorable interaction points, referred to as site points, which are used to define potential binding pockets. For each detected pocket, SiteMap produces contour representations describing the physicochemical characteristics of the cavity, including hydrophobic regions and several classes of hydrophilic regions. The latter are further categorized according to their propensity to participate in hydrogen-bond donation, hydrogen-bond acceptance, or metal coordination interactions. To estimate the suitability of each cavity for ligand binding, two scoring metrics were calculated: SiteScore (Equation 1) and DScore (Equation 2). In these equations, *n* denotes the number of site points, *p* represents the hydrophilic score, and *e* corresponds to the enclosure score.

$$\text{SiteScore}=0.0733\sqrt{n}+0.6688e-0.20p$$ (1)

$$\text{DScore}=0.094\sqrt{n}+0.6688e-0.324p$$ (2)

SiteScore is primarily used to rank predicted cavities and identify the most probable ligand-binding region within a protein structure. Values equal to or greater than 0.80 generally indicate a ligand-binding site, whereas scores above 1.01 are associated with highly favorable pockets. DScore provides an estimate of cavity druggability and is commonly used to compare binding sites across different proteins. Based on this metric, pockets are classified as druggable (DScore > 0.98), difficult or moderately druggable (0.83–0.98), or undruggable (DScore < 0.83). Scores approaching 1.0 for either metric suggest a binding site with promising characteristics for small-molecule recognition.

Blind docking experiments were subsequently carried out using CB-Dock2 (Liu et al., 2022). This automated docking platform combines cavity detection with ligand docking, allowing binding-site prediction without prior knowledge of the interaction region. The method employs a template-guided strategy to improve both pocket identification and ligand pose prediction and has been reported to achieve docking accuracies frequently associated with root-mean-square deviation (RMSD) values below 2.0 Å.

### 2.3 Molecular docking

The molecular docking studies were performed using the Glide v9.0 Schrödinger Suite 2024-1 [Schrödinger, LLC, New York, NY, United States)], utilizing the extra precision (XP) (Friesner et al., 2006) mode and the OPLS4 force field. The grid box was constructed on the site points of the sites identified in SiteMap. The study was performed according to the settings previously used (Culletta et al., 2020; Culletta et al., 2022).

### 2.4 Binding pose metadynamics (BPMD)

Binding Pose Metadynamics (BPMD) is an enhanced-sampling protocol based on metadynamics that enables automated evaluation of ligand binding poses. By applying metadynamics biasing forces, the ligand is encouraged to explore conformational space around its binding mode during molecular dynamics simulations. This method has demonstrated the ability to identify native-like binding poses and to effectively re-rank poses generated through docking or induced-fit docking studies (Clark et al., 2016).

In this study, 10 independent BPMD simulations of 10 ns each were carried out, employing the root-mean-square deviation (RMSD) of the ligand heavy atoms as the collective variable (CV). Prior to RMSD calculation, protein residues located within 3 Å of the ligand were selected for structural alignment. The Cα atoms of the binding-site residues were then aligned to the corresponding atoms in the initial frame of the metadynamics trajectory. Subsequently, the heavy-atom RMSD of the ligand relative to its initial conformation was determined (Fusani et al., 2020).

To assess the stability of the ligand throughout the simulations, three BPMD-derived metrics were calculated at the end of each trajectory: the PoseScore, PersistenceScore (PersScore), and CompositeScore (CompScore) (Allegra et al., 2021; Culletta et al., 2021).

### 2.5 Molecular dynamics and molecular mechanics-generalized born surface area (MM-GBSA)

Three plain MD simulation replicas of 500 ns each were carried out using Desmond 4.9 [Schrödinger Suite 2024-1 (Schrödinger, LLC, New York, NY, United States)] (Bowers et al., 2006) with the OPLS4 force field. The complexes were solvated in cubic boxes using the TIP3P water model. Three Na^+^ were added to neutralize charges. The temperature of the systems was set to 303.15 K, and the pressure of the systems was set to 1.013 bar. The system was simulated as an NPT ensemble with a Nose-Hover thermostat and a Martyna-Tobia-Klein barostat. The trajectories frames were clustered according to the hierarchical cluster linkage method. One thousand frames recorded in each simulation were clustered considering the binding site conformations into clusters based on the atomic RMSDs. The MM-GBSA approach (Genheden and Ryde, 2015) was applied to the snapshots extracted from the 500 ns production MD trajectories. The free energy of protein-ligand binding using MM-GBSA was calculated as the difference between the energy of the bound complex and the energy of the unbound protein and ligand (Jacobson et al., 2004). The settings were previously reported in de Carvalho et al. (2023), Culletta et al. (2021), and Culletta et al. (2020).

### 2.6 Virtual screening libraries

The FDA and DrugBank libraries were used as sources to perform virtual screening. Once downloaded, the compounds within the library were optimized using the same protocol described above.

### 2.7 Cell cultures and chemical compounds

Hela (Masone et al., 2023) and GCIY (RCB0555, Riken) cell-lines were cultured in DMEM/F12 and RPMI medium (Euroclone, Pero, Italy) supplemented with glutamax, penicillin (100 units/mL)/streptomycin (100 μg/mL) and 10% fetal bovine serum (Euroclone, Pero, Italy). Chemical compounds including ticagrelor (CAS: 274693-27-5), glyburide (CAS: 10238-21-8), aliskiren (CAS: 173334-57-1), nitazoxanide (CAS: 55981-09-4), lansoprazole (CAS: 103577-45-3), pentostatin (CAS: 53910-25-1), lapatinib (CAS: 231277-92-2), and ribavirin (CAS: 36791-04-5) were purchased from Cayman Chemical (Ann Arbor, Michigan 48108, United States) and dissolved in DMSO.

### 2.8 Luciferase assays

For luciferase assays, a mammalian NR2F2 reporter was constructed by inserting in the pNL2.1plasmid (Promega, Madison, United States) an NR2F2 binding site found in the NGF1A promoter. The following sequences were used (FW 5‘- tcgag TCA CGG CGG AGG CGG GCC CGG GTA TAT AA a -3’, RV 5‘- ag ctt TT ATA TAC CCG GGC CCG CCT CCG CCG TGA c -3’) (Wang et al., 2021). pcDNA3.1 NR2F2_HA and pcDNA3.1 NR2F6_FLAG plasmids expressing full lenght NR2F2 (NM_021005.4) and NR2F6 (NM_005234.4) were purchased from GenScript (Piscataway, New Jersey, United States). A NR2F2 delete derivative lacking NR2F2 ligand-binding domain and encompassing aa 1-177 of NR2F2 was obtained by subcloning in pcDNA3.1 a PCR amplicon generated using the following primers: NR2F2 LBD FW 5′-CAT​AAG​CTT​ATG​GCA​ATG​GTA​GTC​AGC​ACG-3'; NR2F2 LBD RV 5'-CAT​AAG​CTT​GTA​CGA​GTG​GCA​GTT​GAG​GGG-3′). Hela cells were seeded in 96 well plates in the presence of DMEM/F12 media supplemented with 10% FBS. Cells were co-transfected with expression vectors for the NR2F transcription factors, and the NanoLuc reporter construct along with a Firefly luciferase plasmid as an internal control (pGL4.54_Luc2/TK). 18 h post-transfection, cells were treated with the indicated compounds for 24 h. Cells were lysed according to the Nano-Glo Luciferase assay guidelines (Promega, Madison, United States). Two replicates were performed for each experimental point. Luminescence was measured in a GloMAx microplate reader.

### 2.9 Proliferation assays

Sulforhodamine B (SRB) Assay (Voigt, 2005) or CellTiter-Glo® Luminescent Cell Viability Assay (Promega, Madison, United States) were used to assess the effect of the analyze compounds on cancer cell lines. For Sulforhodamine B (SRB) Assay, cells were seeded in 96-well plates (130,000 cells/mL) and treated with the compound of interest at various concentrations. After 72 h, cells were fixed in 10% trichloroacetic acid (TCA, MERCK, Darmstadt, Germany). Cells were then washed and stained with 4% Sulforhodamine B (MERCK, Darmstadt, Germany). The protein-bound dye was subsequently solubilized with 10 mM Tris buffer (pH 10.5) and well absorbance measured at 540 nm using a spectrophotometer (Tecan Infinite 200). Cell viability assessed using the CellTiter-Glo® Luminescent Cell Viability Assay (Promega, Madison, United States) was performed according to the manufacturer’s instructions. ATP-dependent luminescence was measured 72 h after compound addition to the cell cultures using a GloMax microplate reader (Promega, Madison, United States). Statistical analyses were performed using GraphPad Prism software. Data are presented as the mean ± standard deviation (SD). Differences in cell growth between two independent groups were evaluated using a two-tailed unpaired Student’s t-test. Statistical significance was defined as *p* < 0.05.

### 2.10 RNA interference experiments

GCIY gastric cancer cells were silenced using a pool of two validated siRNA (Ambion™ Silencer™ Select siRNAs, oligo ID s14021 and s14023, Thermo Fisher Scientific, Waltham, Massachusetts, U.S.) against NR2F2 and a control siRNA without target sequence (Ambion™ Silencer™ Select Negative Control siRNAs, cat. N° 4390843 and 4390846, Thermo Fisher Scientific, Waltham, Massachusetts, U.S.). Reverse transfection in 96-well plates was performed using Lipofectamine RNAiMAX (Thermo Fisher Scientific, Waltham, Massachusetts, U.S.) according to the manufacturer instructions to achieve a final concentration of 12 nM siRNA. Cells harvested in log phase were resuspended in antibiotic-free complete medium and seeded directly onto the complexes. Gene silencing efficiency was evaluated by Western immunoblot analysis of samples collected 48 h post-transfection.

### 2.11 Western blot analyses

Western blot experiments were performed as described (Zanetti et al., 2015). Briefly, protein lysates from cell cultures were obtained after lysis in RIPA buffer (NaCl 150 mM, Igepal 1%, Sodium deoxycholate 0.5%, SDS 0.1%, Tris-HCl, pH 7.5) supplemented with protease inhibitors (Protease Inhibitor Cocktail, EDTA free, MCE) and/or PhosSTOP Phosphatase Inhibitor Cocktail, Merck, Darmstadt, Germany). Cell lysates were separated by SDS-PAGE and finally transferred to nitrocellulose membranes. Membranes were incubated overnight in TBST (NaCl 150 mM, TrisHCl 20 mM, Tween 20 0.1%) plus 5% BSA with the following antibodies: anti-NR2F2 (Cell Signalling, Danvers, Massachusetts, 01923, United States), anti-tubulin (Sigma, Merck, Darmstadt, Germany). Blots were subsequently incubated with Cy5-conjugated goat anti-rabbit (Jackson ImmunoResearch, West Grove, Pennsylvania 19390, United States) or Cy-3 goat anti-mouse (Jackson ImmunoResearch, West Grove, Pennsylvania 19390, United States) for 1 h at room temperature and analyzed using an automated fluorescence scanner (Typhoon, GE Healthcare, Chicago, Illinois, United States).

## 3 Results

### 3.1 Structure refinement, SiteMap and blind docking

**FIGURE 2:**
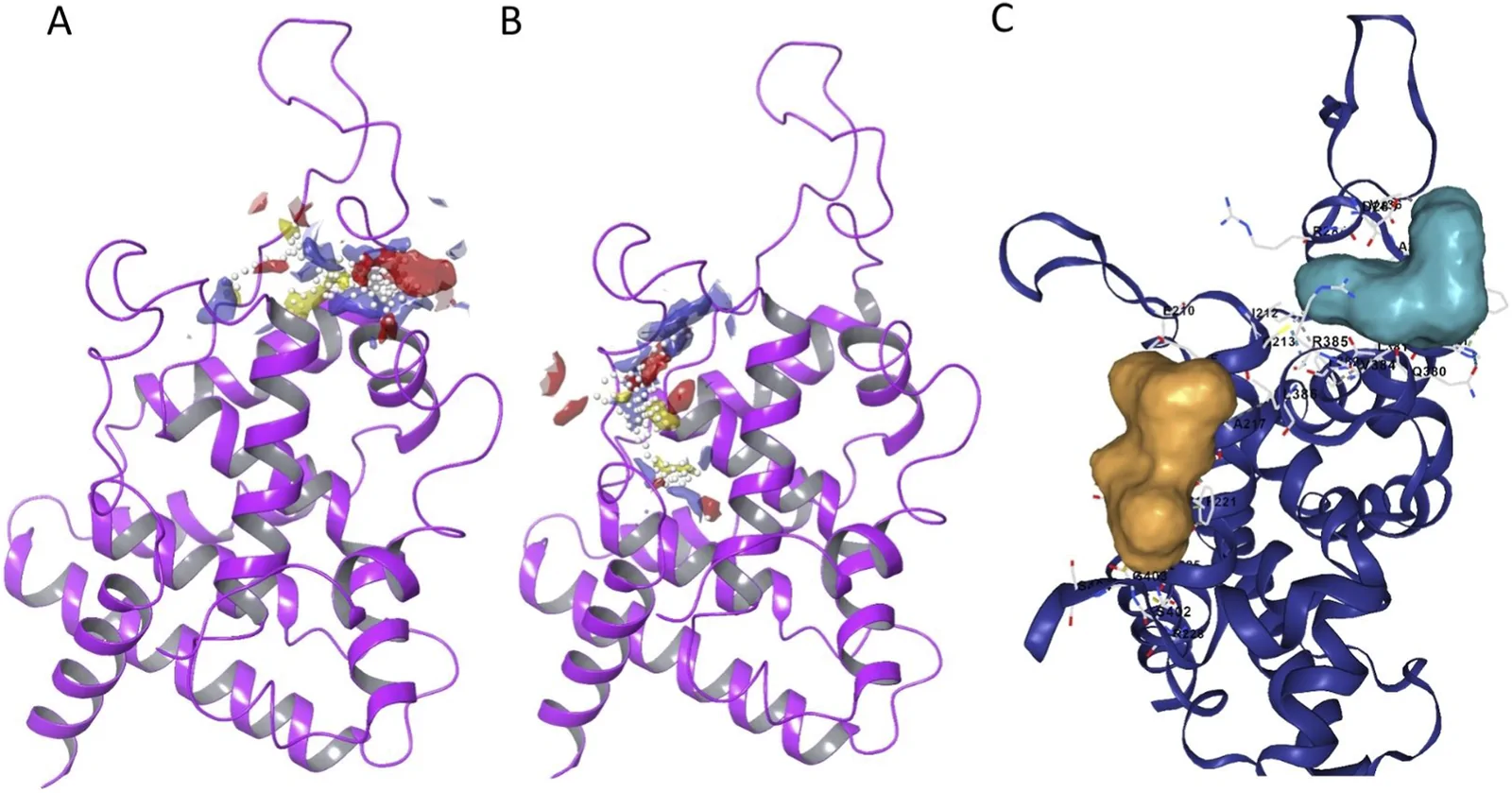
Site mapping results on the ligand binding domain. Site 1 **(A)**, Site 2 **(B)**, and sites identified with the blind docking analysis Site 1 in blue and Site 2 in orange **(C)**.

The unique crystal apo-structure of the human COUP-TFII ligand binding domain (PDB ID: 3CJW) was selected to start computational studies (Figure 2). First, the crystal structure was optimized by completing and refining the missing loops, adding Thr193–Met207 and Pro268-Val286 residues, and optimizing the amide groups of asparagine (Asn) and glutamine (Gln) and the imidazole ring in histidine (His); and predicted the protonation states of histidine, His, aspartic acid (Asp) and glutamic acid (Glu) and the tautomeric states of histidine. At the same time, ligand CIA1 was prepared. A site mapping was performed all over the surface of the ligand binding domain to identify potential binding sites. Two potential binding sites, designated Sites 1 and 2, were selected. As illustrated in Figures 2A,B, the selected sites exhibit a favorable size (depicted as ‘site point’ white) for druggability, as well as the requisite hydrophobic (yellow), hydrogen-bond donor (blue), and hydrogen-bond acceptor (red) characteristics for potential interactions with a ligand. These include the presence of non-polar groups and hydrogen-bond acceptors, and donors.

Site 1 showed a SiteScore of 1.028, a DScore of 1.063, a volume of approximately 243.53 Å^3,^ and a total solvent accessible surface area of 338.37 Å^2^. The site involves residues Met201-Cys213, Leu215, Pro263-Leu264, Val266-Ala267, Asp283-Phe288, His291-Ile292, Phe295, Gln380-Phe382, and Val384-Arg385. A SiteScore of 1.048, a DScore of 0.964, a volume of 187.96 Å^3^ and a total solvent accessible surface area of 256.06 Å^2^ were returned for Site 2. The residues that characterized the site include Glu189, Pro190-Pro192, Ser194-Arg195, Ser198-Gln199, Cys200-Met201, Ile206-Met207, Ile209-Glu210, Leu215, Arg218-Met219, Ser222-Ala223, Trp226, Asn256, Gln259-Cys260, Lys316, and Leu386.

To confirm the binding sites retrieved after the site mapping analysis, a blind docking analysis was conducted utilizing the CB-Dock2 software with CIA1 as a ligand. As illustrated in Figure 2C, the sites identified through this analysis align with those identified in the site mapping analysis. Site 1 is represented in blue, while Site 2 is shown in orange.

Moreover, we performed an alignment with RXR-gamma (PDB ID:7A79), and even though the receptors have a sequence identity of 40.97%, the binding sites retrieved are quite different as reported in the super positioning in Supplementary Table S1. The binding Site 1 of NR2F2 is the nearest to the RXR binding site, but they are not superimposable.

### 3.2 Molecular docking

**FIGURE 3:**
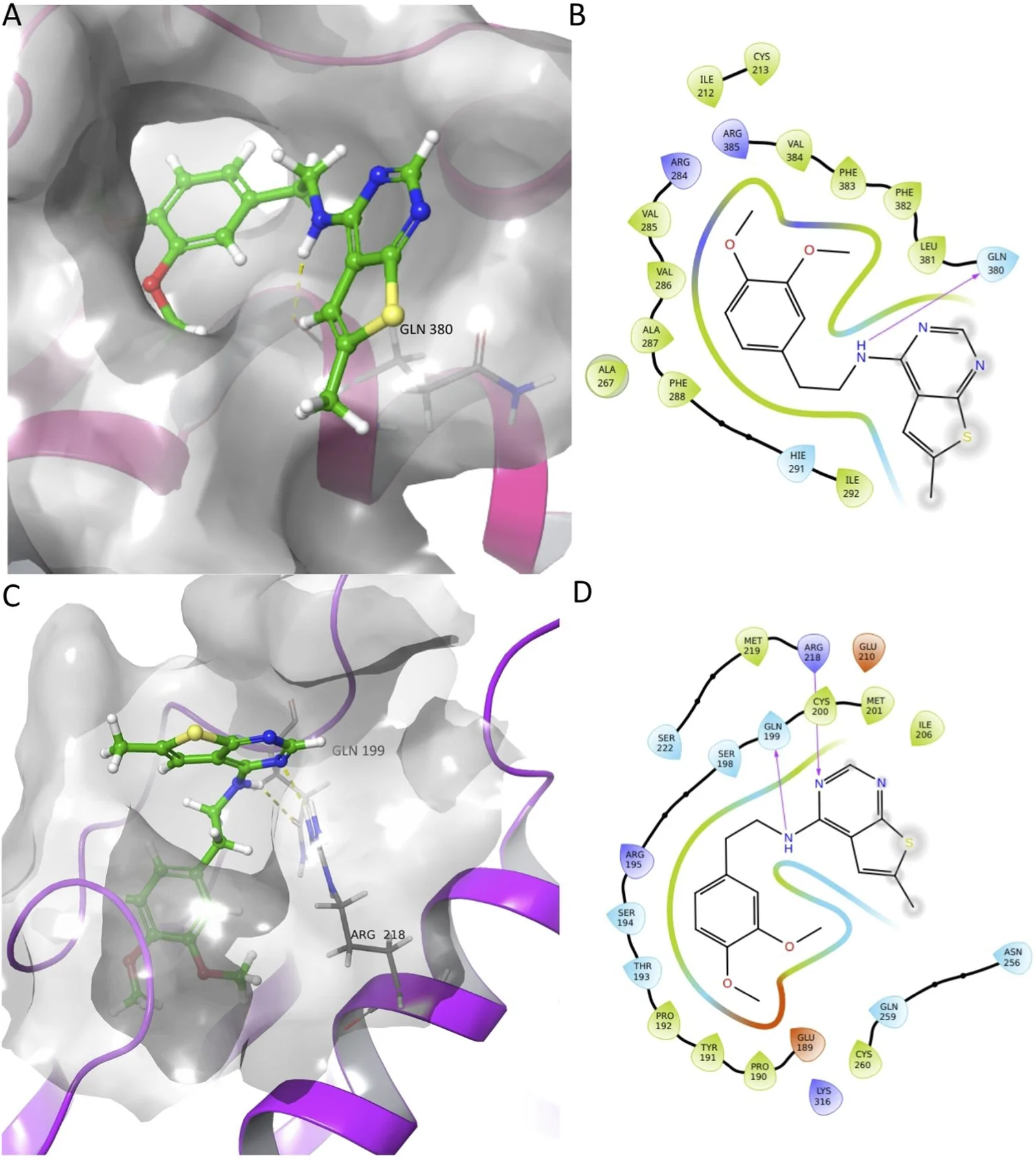
XP binding pose representation and 2D interactions diagrams of the CIA1 in Site 1 **(A,B)** and Site 2 **(C,D)**. The H-bond hydrogen interactions are represented in yellow dashes in 3D binding pose and purple arrows in the 2D interactions diagram.

Based on the results obtained from the site mapping analysis, extra precision (XP) docking studies were conducted to elucidate the binding mode and interactions of CIA1 with the two identified sites. Consistent with the SiteMap and CB-Dock2 predictions, the results showed that CIA1 binds to Sites 1 and 2. It is possible to observe that in Site 1, the dimethoxyphenylethyl portion of the ligand engages with both the hydrophobic and non-polar portions of the binding site. Conversely, the 6-methylthieno [2,3-d]pyrimidine portion of the ligand interacts with the polar portion of the binding site. The only interaction observed involved a hydrogen bond donator between the 4-amine and the carbonyl of Gln380, which is situated on the polar surface of the site (Figures 3A,B). The same behavior was observed in Site 2: the dimethoxyphenylethyl portion of the ligand engaged with both the hydrophobic and non-polar portions of the binding site. Meanwhile, the 6-methylthieno [2,3-d]pyrimidine portion of the ligand interacts with the polar portion of the binding site. In terms of the interactions, the same type of interaction was observed as in Site 1, namely the interaction between the 4-amine and carbonyl of Gln199, and the hydrogen bond acceptor between the nitrogen of the pyrimidine and the amine of Arg218 (Figures 3C,D).

### 3.3 Molecular dynamics

The limitations of docking methods lie in their failure to consider the flexibility of the entire protein, instead of treating the receptor as a rigid body instead. Conversely, the MD simulation technique considers the flexibility of the complex, thereby facilitating a deeper understanding of the binding mode of the ligand and its behavior in a dynamic environment. Accordingly, an extensive study of the binding mode of the molecule was conducted using long plain molecular dynamics simulations. Consequently, the docking poses of CIA1 in the Site 1 and Site 2 were subjected to 1.5 µs molecular dynamics (3x replicas of 500 ns), using different random seeds. The stability of the complexes was observed through the calculated Root Mean Square Deviation (RMSD) plots and Root Mean Square Fluctuation (RMSF). All protein frames were first aligned to the reference frame backbone and then the RMSD is calculated based on the atom selection, in these cases the Cα (Carollo et al., 2023). The RMSD values of the Cα atoms of the protein and ligand are reported.

#### 3.3.1 Stability analysis

**FIGURE 4:**
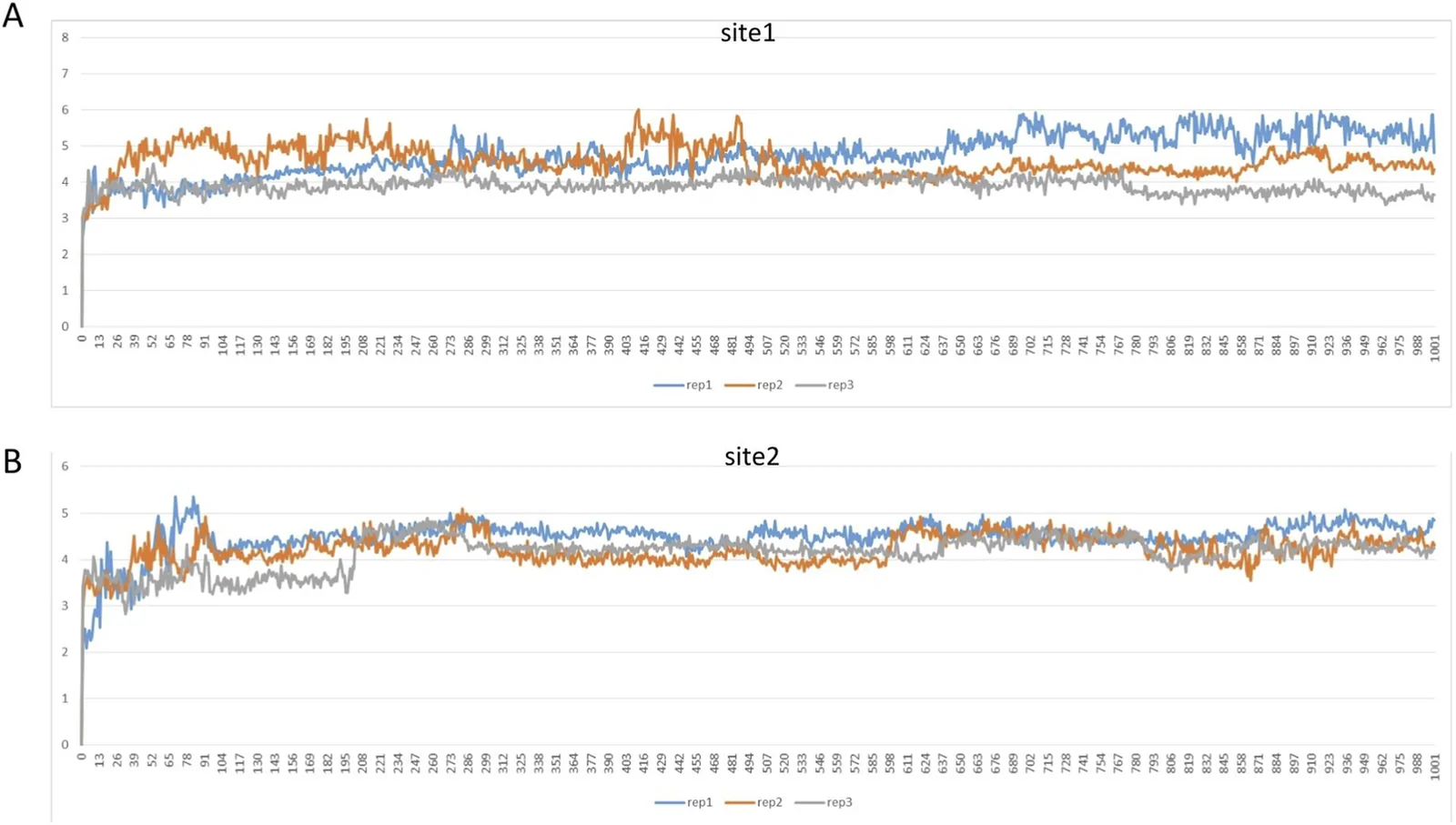
RMSD of NR2F2 ligand-binding domain-CIA1 (**A**, Site 1) and (**B**, Site 2) after 1.5 µs MD simulations. The x-axis represents the simulation frames, and the y-axis represents the protein RMSD (Å).

Following a series of fluctuations, the systems exhibited an RMSD of approximately 4.4 Å for the system with CIA1 bound to Site 1 and 4.0 Å for the system with CIA1 bound to Site 2 (see Figures 4A,B). Both systems maintain stability in a range of 2 Å after a brief period of equilibration in the first 50 ns of the simulations. This first analysis confirms the reliability of the potential identified binding sites.

**FIGURE 5:**
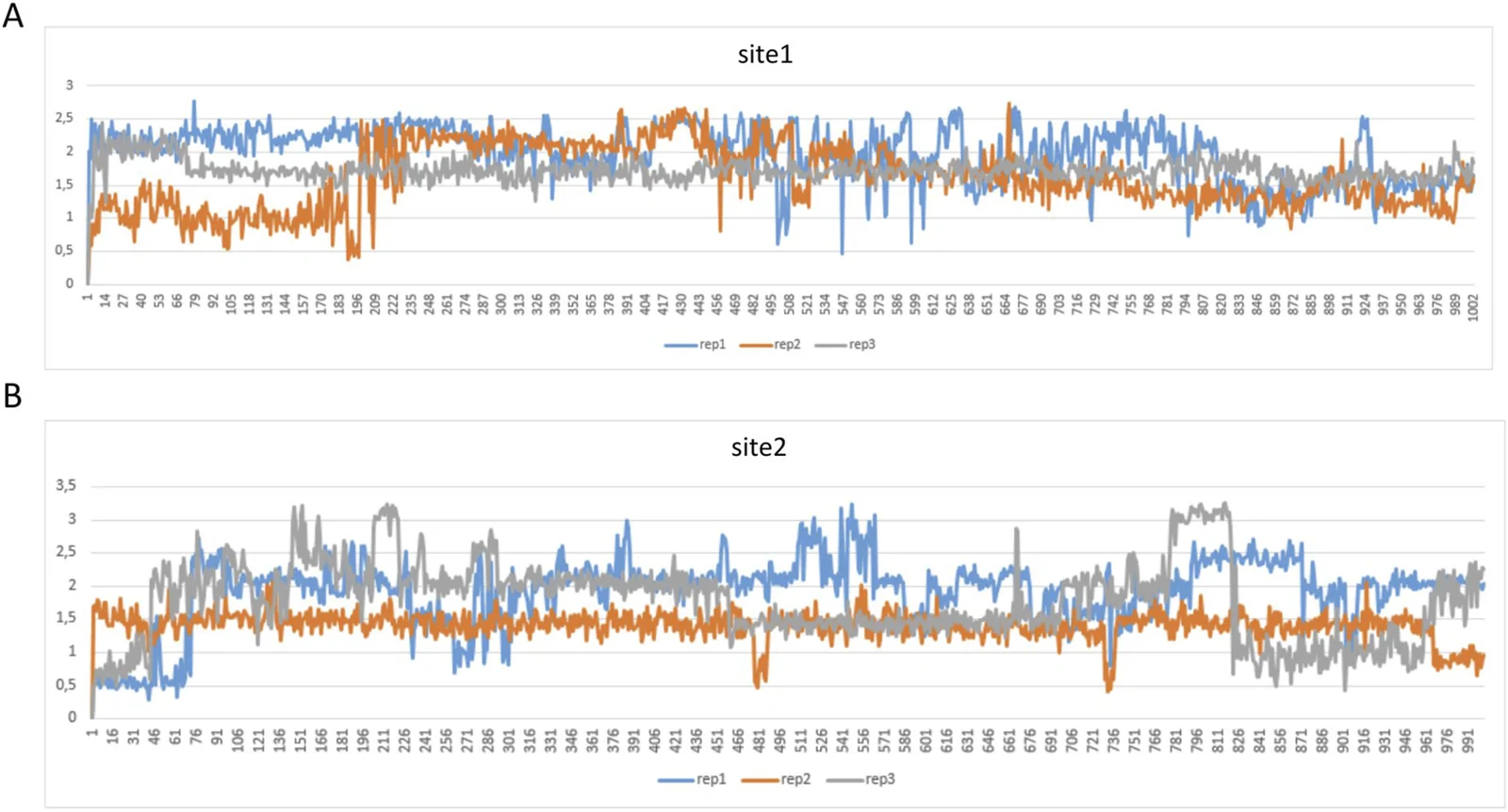
Ligand-RMSD (**A**, Site 1) and (**B**, Site 2) after 1.5 µs MD simulations. The x-axis represents the simulation frames, and the y-axis represents the ligand RMSD (Å).

The ligand RMSD shows the stability of the ligand with respect to the protein and the binding pocket, and if the values observed are significantly larger than the RMSD of the protein, it is likely the ligand detaches from its initial binding site. As reported in Figures 5A,B, the ligand maintains a pose like the starting one moving in all the replicas with an average value of 2 Å. From this point of view, both sites are reliable for the binding of CIA1.

#### 3.3.2 Residues mobility analysis

**FIGURE 6:**
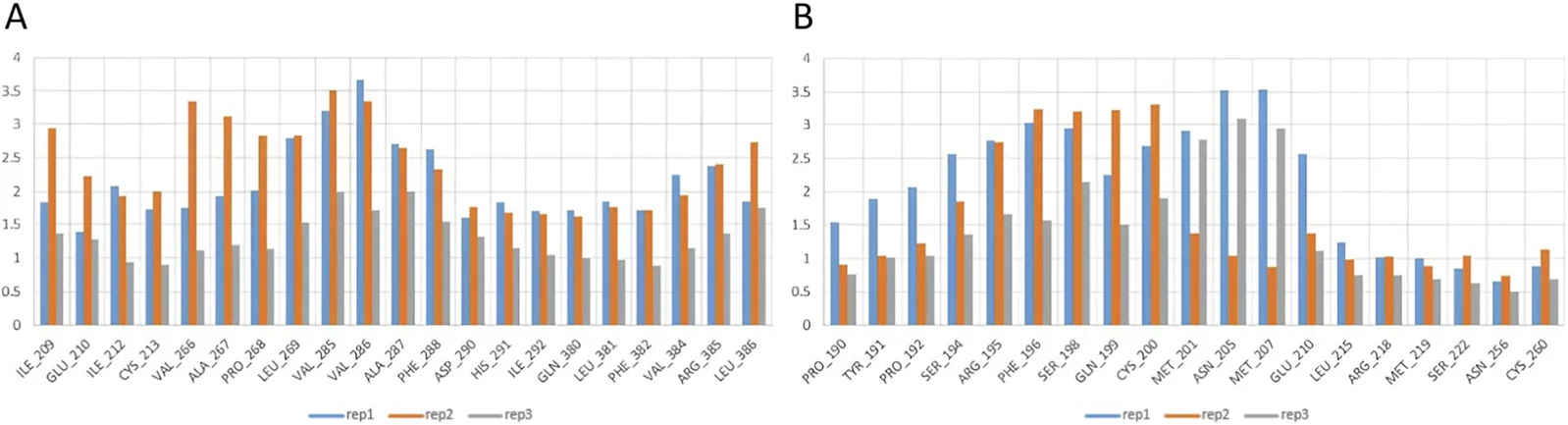
RMSF of NR2F2 ligand-binding domain-CIA1 (**A**, Site 1) and (**B**, Site 2). The x-axis represents the residues, and the y-axis represents the protein RMSF (Å).

The results of the residue mobility (RMSF) analysis were examined with a particular focus on the residues that constitute binding sites. This analysis revealed that the average RMSF values, calculated across the three independent MD replicas, were below 2 Å for residues within both binding sites, indicating limited flexibility and suggesting a stabilizing effect of CIA1 contacts on the backbone structure (Figures 6A,B). Notably, the average RMSF values of these residues on Site 2 are lower than 1 Å: Leu215 (0.99 Å), Arg218 (0.93 Å), Met219 (0.86 Å), Ser222 (0.84 Å), Asn256 (0.63 Å) and Cys260 (0.90 Å). It is of particular interest to note that Arg218 and Ser222, which were previously identified in the docking study, are among these residues.

**FIGURE 7:**
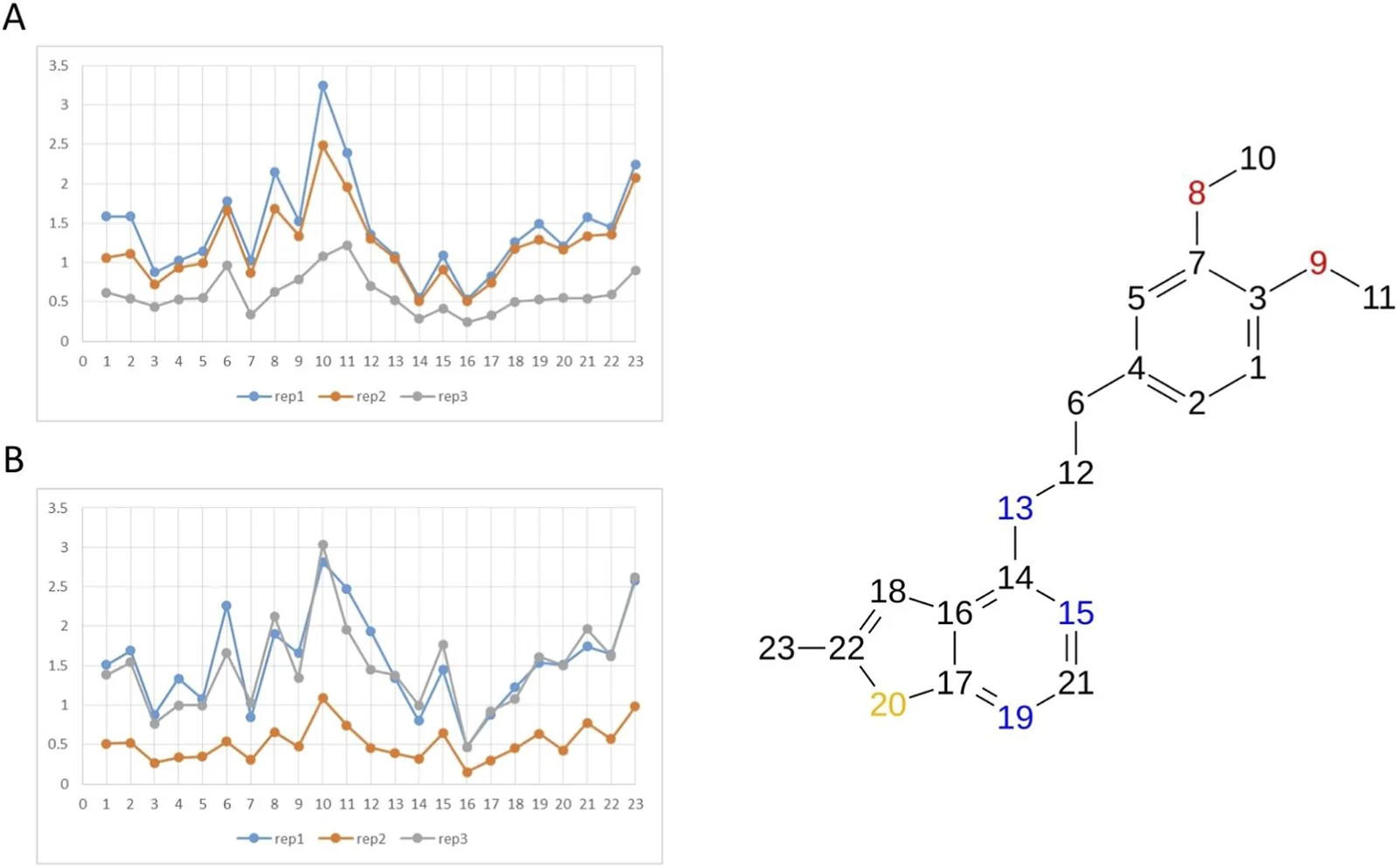
RMSF of ligand (**A**, Site 1) and (**B**, Site 2) after 1.5 µs MD. The x-axis represents the ligand atom index, corresponding to the atom numbering shown in the ligand structure displayed alongside the plot, while the y-axis represents the ligand RMSF (Å).

In Figure 7, the ligand RMSF plot demonstrated that the dimethoxyl groups and the linker exhibited the greatest fluctuations among the ligand substructures in each site, with an RMSF value reaching between 1 and 3 Å. It is noteworthy that most fluctuations observed in Site 1 occur within the loop region encompassing residues Val266 to Phe288, whereas in Site 2, the fluctuations are predominantly concentrated within the loop comprising Ser194 to Gln199. These fluctuations were a consequence of the ligand shift that emerged during the visual inspection of the trajectories.

**FIGURE 8:**
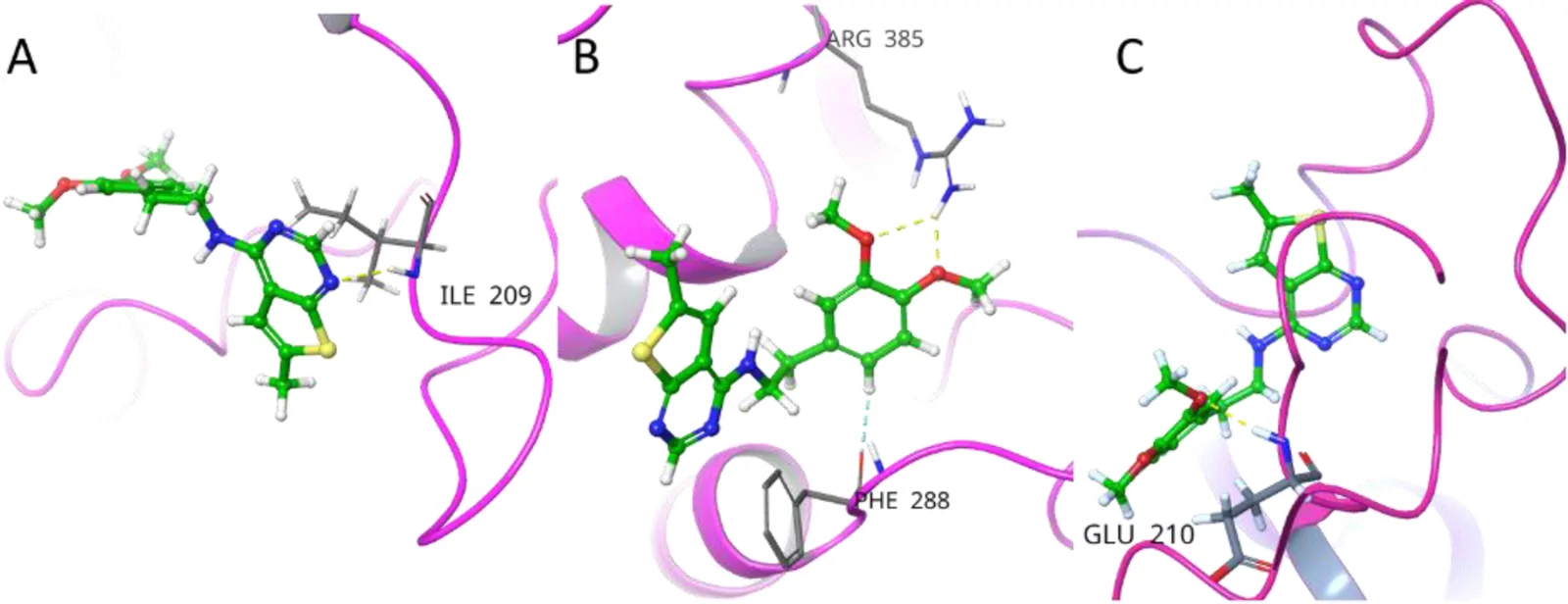
The interactions observed during the three replicas of the CIA1 in Site 1 **(A–C)** and Site 2 **(D–F)**. The yellow dashes represent H-bond interactions, and light blue dashes the aromatic interaction.

As previously mentioned, the visual inspection of the trajectory for Site 1 revealed that CIA1 shifted from its original docking position (H-bond with Gln380) and established new interactions, such as H-bond and hydrophobic interactions with Ile209, Glu210, Ala267 and water-bridge-mediated H-bond interactions with Arg385. Additionally, several aromatic bonds were observed between the thienopyrimidine group and Phe288 and π-π contacts with dimethoxy groups and Glu210 (Figures 8A–C; Table 1 and Supplementary Table S2).

**TABLE 1:** Target–ligands interactions observed in the docking analysis and in the MD simulations.

​	Docking		MD					
			Replica 1		Replica 2		Replica 3	
Site	Residue	Int.*	Residue	Int.*	Residue	Int.*	Residue	Int.*
1	Gln380	HB	**Ile209**	WB	**Ile209**	Hphob	**Ile209**	Hphob, WB
			**Ala267**	HB, Hphob, WB	Glu210	HB	Ile212	Hphob
			Arg284	WB	Arg284	HB, Hphob, WB	**Ala267**	HB
			Phe288	Hphob	His291	HB	Val384	Hphob
			Gln380	WB	​	​	**Arg385**	WB
			**Arg385**	HB, Hphob, WB	​	​	​	​
2	Gln199	HB	**Tyr191**	Hphob	**Tyr191**	Hphob	**Tyr191**	Hphob
	Arg218	HB	Phe196	Hphob	**Arg218**	WB	Pro192	HB, WB
			**Arg218**	HB, Hphob, WB	**Ser222**	HB	Ser194	WB
			**Ser222**	HB, WB	Cys260	Hphob	Gln199	HB, WB
							Glu210	WB
							**Arg218**	WB

Int.* is for interactions, HB is for H-bonds, vdW is for van der Waals interactions, Hphobic is for hydrophobic interactions, and WB is for water bridges. Bold values indicate amino acid residues shared in common. “Res” refers to an amino acid residue.

In the docking study conducted on Site 2, the molecule was observed to interact with Gln199 and Arg218 via hydrogen bonds. The interactions with Arg218 are conserved during the three replicas of the MD simulations. Another interaction conserved during all replicas is the hydrophobic one with Tyr191. Notably, in rep1, in addition to the formation of a hydrogen bond with Arg218, the molecule engaged in both hydrophobic and water bridge interactions with the same residue. The remaining two replicas, rep2 and rep3, corroborated the water bridge interaction by Arg218 observed in rep1. Furthermore, in rep1 and rep2, the interaction with Arg218 is reinforced by hydrogen bond interactions with Ser222. (Figures 8D,E; Table 1 and Supplementary Table S2).

It is interesting to note that in replica 1 and replica 2, the ligand did not change its starting orientation but only moved slightly, allowing the dimethoxy moiety to be accommodated deeper into the binding pocket and leading to better accommodation of the molecule. In replica 3, the ligand orientation was completely inverted at the end of the simulation. However, this has not led to any major changes in the RMSD and RMSF values.

**FIGURE 9:**
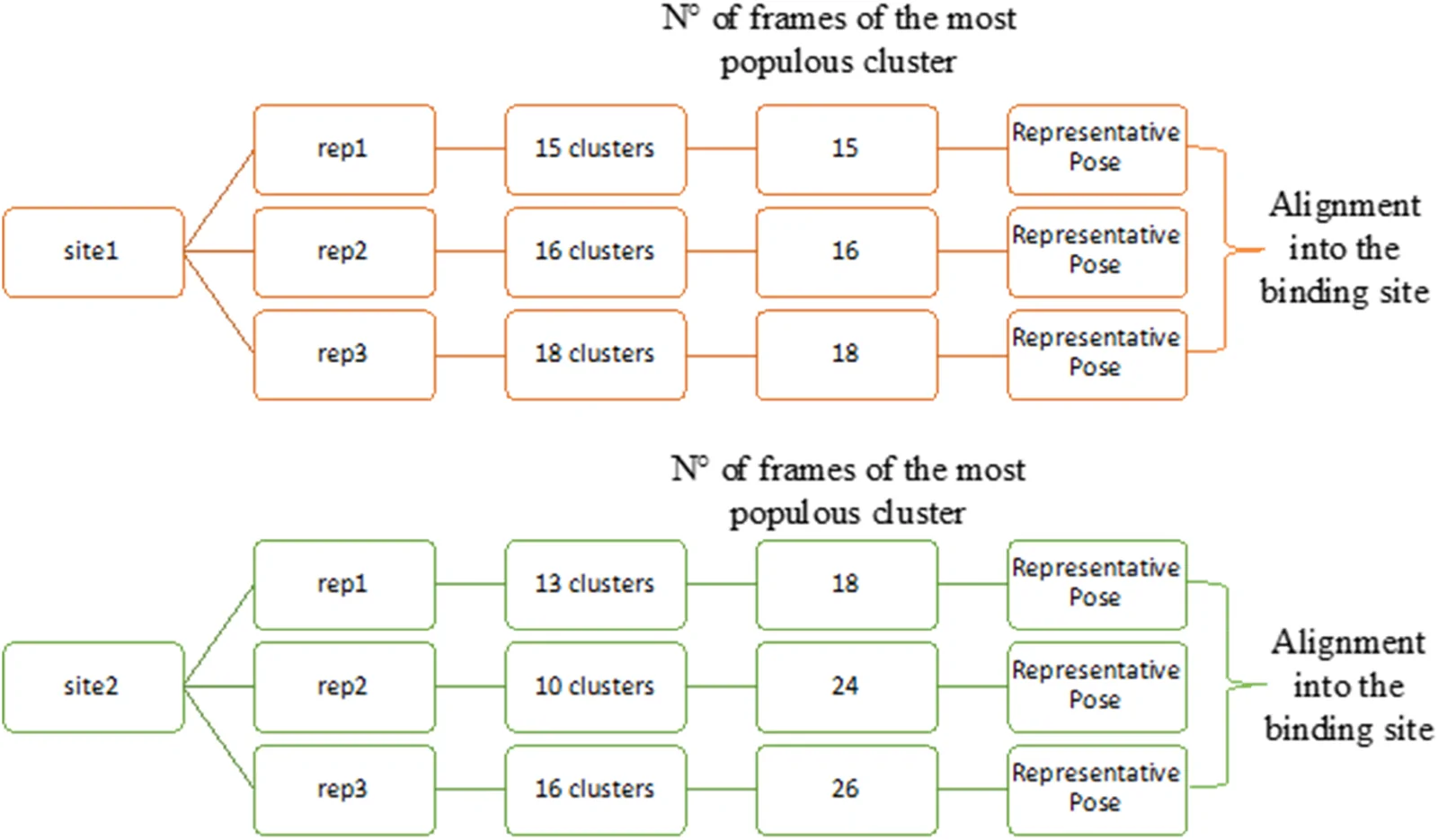
Diagram of the trajectory cluster analysis of Site 1 and Site 2.

**FIGURE 10:**
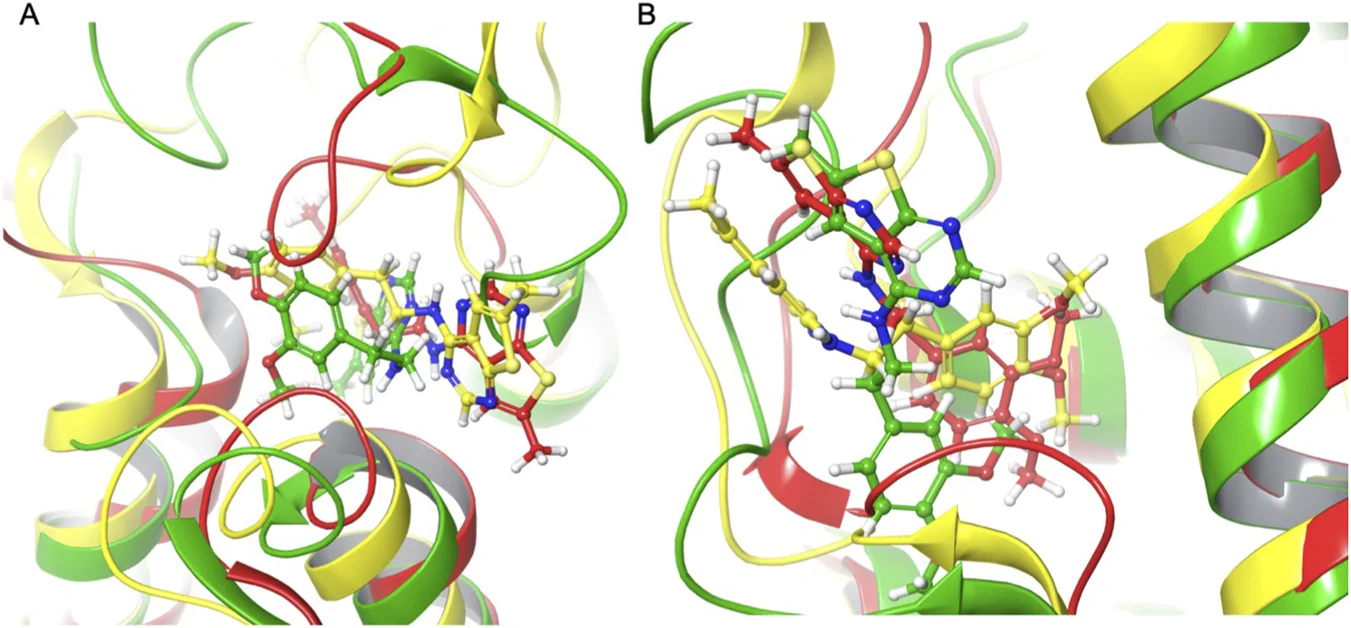
Superposition of the representative trajectory cluster analysis in Site 1 **(A)** and Site 2 **(B)**. Protein-ligand in red is rep1, in yellow rep2, and in green rep3.

To corroborate these results, we decided to perform a trajectory cluster analysis for each site and for each MD replica. Each trajectory, comprising 1,000 recorded frames, was clustered based on the root mean square deviation of the binding site backbones, resulting in more representative clusters. The most populated clusters from each replica were subsequently selected and aligned to evaluate whether CIA1 maintained a conserved binding mode within the binding sites across the independent simulations. As shown in Figure 9, for Site 1, we obtained 15 clusters for replica 1, 16 for replica 2, and 18 for replica 3. Similarly, for Site 2, we identified 14 clusters for replica 1, 10 for replica 2, and 16 for replica 3. The alignment of the observed representative poses for each site showed that CIA1 maintained its binding in both active sites (Figure 10).

### 3.4 Binding pose metadynamics (BPMD)

Subsequently, a binding pose metadynamics protocol was employed to assess the consistency of CIA1 docking poses within the previously identified binding sites. The pose stability is evaluated by using three different scores: PoseScore, PersScore, CompScore. BPMD was performed on the docked pose and the last frame (500 ns) of the classical MD simulations representing the equilibrated ligand pose. Low values indicate more stable complexes.

The results from BPMD demonstrated that for Site 1 a PoseScore of 4.063 and 1.216 - 1.746 for the original docked pose and the last MD frames, respectively (Figures 10A,B). PoseScore should be ≤ 2 Å to consider the ligand poses stable. The PoseScore obtained during the MD simulations shows that the poses are more reliable when compared to the starting docked pose, thus confirming the output from the plain MD simulations, which showed a slight shift of the ligand during the run. The persistence of the interactions was found to increase, as evidenced by PersScore values of 0.136 and 0.364-0.827 for the original docked pose and the final MD poses indicate that the H-bond interactions registered during the MD replicas (Glu210, Met289, Phe288 and Arg385) stabilized the complex. The CompScore for the original pose and the last frame MD poses of the hit ligand were found to be 3.381 and −1.238, respectively, to confirm better stability.

**FIGURE 11:**
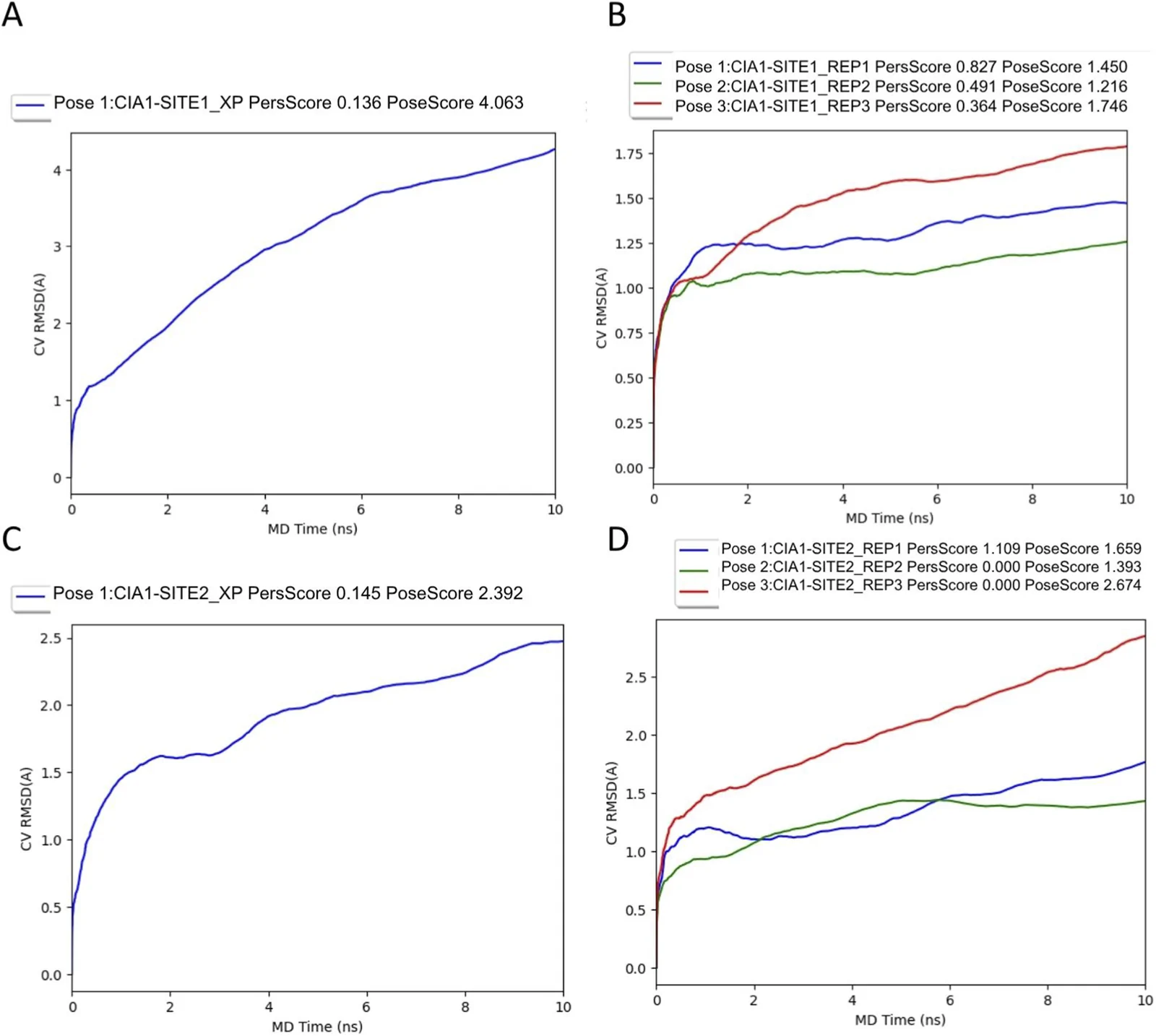
Plots of the RMSD estimate averaged over all 10 trials vs. the simulation time for the BPMD runs. **(A)** the blu line represents the mean CV RMSD values of the XP docking study of the complex Site 1-CIA1. **(B)** blue, red, and green lines represent the mean CV RMSD of the last frames of three replicas of MD of the complex Site 1-CIA1. **(C)** the blu line represents the mean CV RMSD values of the XP docking study of the complex Site 2-CIA1. **(D)** blue, red, and green lines represent the mean CV RMSD of the last frames of three replicas of MD of the complex Site 2-CIA1).

A PoseScore of 2.394 and 1.393 - 2.674 for the original docked pose and the last MD frames, respectively, was calculated for Site 2 (Figures 11C,D). The result of the persistence of the interaction showed an increase in the MD poses with a PersScore of 0.145 and 1.109 for the original docked pose and the last MD frame pose, respectively. In particular, the H-bond of Ser222 registered during the MD stabilized the complex. The CompScore for the original pose and the last frame pose of the hit ligand were found to be 1.665 and 2.674 respectively.

Based on these values obtained, the interactions obtained from the molecular dynamics (MD) simulations demonstrated a higher stability of the two sites compared to those from docking studies. This suggests that the Glu210, the Ala267, and the Arg385, which form hydrogen bonds, as well as the Ile209, which forms a water bridge or participates in hydrophobic interactions, may play a pivotal role in the binding of other molecules in Site 1. Similarly, regarding Site 2, the Ser222 residue, which forms a hydrogen bond, and the Arg218 residue, which participates in a water bridge or hydrophobic interactions, are of particular interest.

### 3.5 Molecular mechanics-generalized born surface area (MM-GBSA)

**FIGURE 12:**
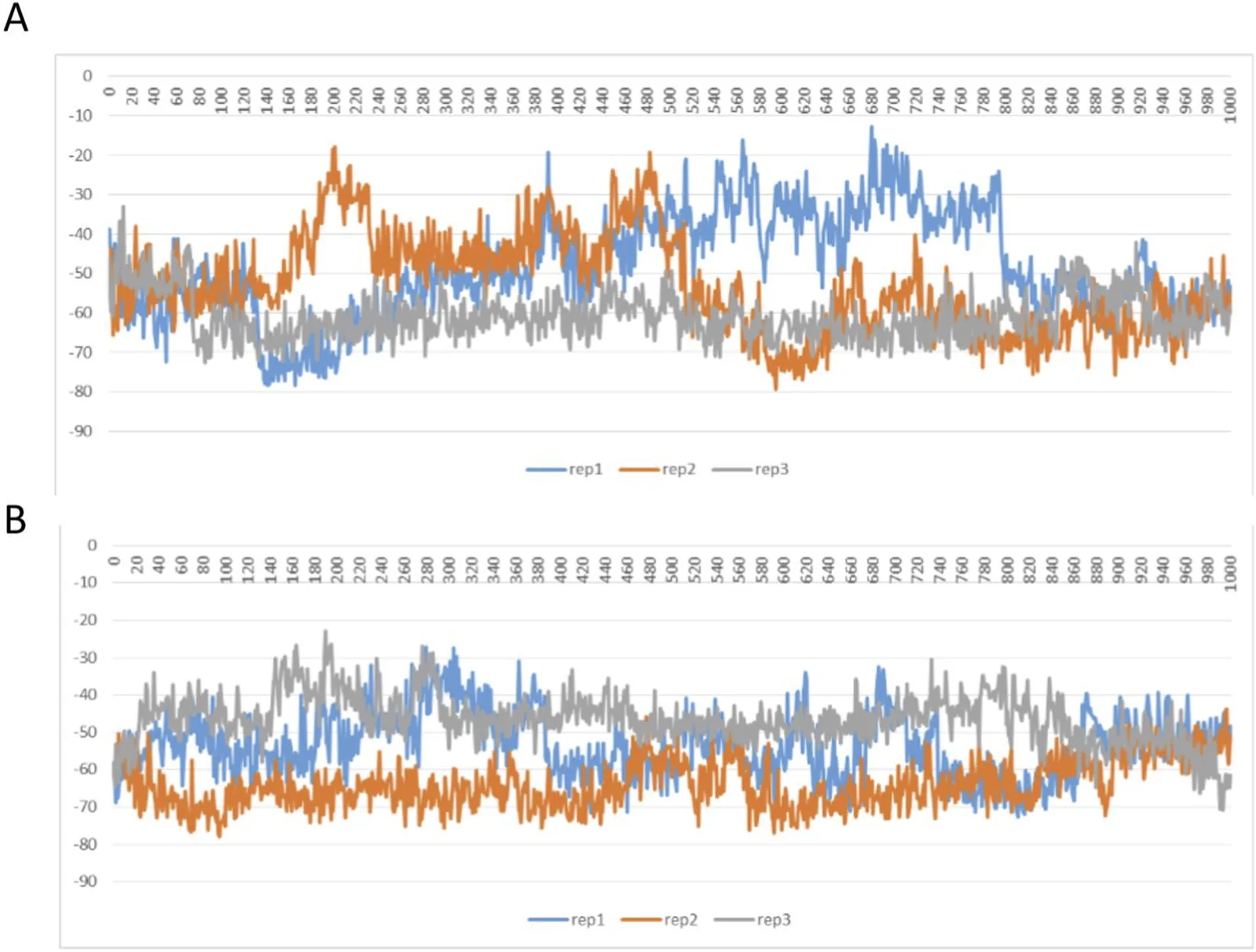
The binding energy (ΔG expressed in kcal/mol) plot of the complex Site 1 **(A)** and Site 2 **(B)**. The x-axis represents the trajectory frames, and the y-axis represents the free energies (ΔG in kcal/mol).

A Molecular Mechanics-Generalized Born Surface Area (MM-GBSA) approach was subsequently applied to each trajectory of the Molecular Dynamics (MD) simulations. This approach offers a more comprehensive insight into the contribution of specific energy terms to the inhibitory potency, including ΔGvdW, ΔGCoul, ΔGHbond, and ΔGLipo. For the calculation of ΔGbind and the individual contributions, 1,001 frames were extracted from each MD trajectory. As anticipated based on the preceding analysis and the available experimental evidence, CIA1 demonstrated a binding affinity for both sites, with ΔGbind average value of −54.103 kcal/mol for Site 1 and slightly higher ΔGbind average value of −58.364 kcal/mol for Site 2. The primary contribution to the binding was provided by the van der Waals energy (ΔGvdW) ∼ −40 kcal/mol and the London dispersion energy (ΔGlipo) ∼ −17 kcal/mol. These energies result from the two aromatic rings (thienopyrimidin and dimethoxyphenyl) of the molecule occupying the hydrophobic pocket of the two sites. Table 2 and Figure 12 present a summary of the binding components in the binding and free energy plot of CIA1 in Sites 1 and 2. Interestingly, the binding energy in Site 2 shows a more stable trend than in Site 1 reflecting the behavior noted in the Ligand RMSD.

**TABLE 2:** Predicted MM-GBSA average free energies (kcal/mol), individual energy terms and standard deviations (σ)of the CIA1-Site 1 and CIA1-Site 2.

​	ΔGbind	ΔGCoul	ΔGHbond	ΔGLipo	ΔGSolv	ΔGvdW
CIA1/Site 1	−54.103 (σ 6.326)	−6.894 (σ 2.005)	−0.595 (σ 0.110)	−17.454 (σ 2.233)	13.863 (σ 0.579)	−41.231 (σ 5.229)
CIA1/Site 2	−58.364 (σ 7.233)	−7.732 (σ 2.859)	−0.589 (σ 0.151)	−18.447 (σ 1.986)	14.952 (σ 1.421)	−40.524 (σ 4.193)

### 3.6 Virtual screening and FDA compounds repurposing

**FIGURE 13:**
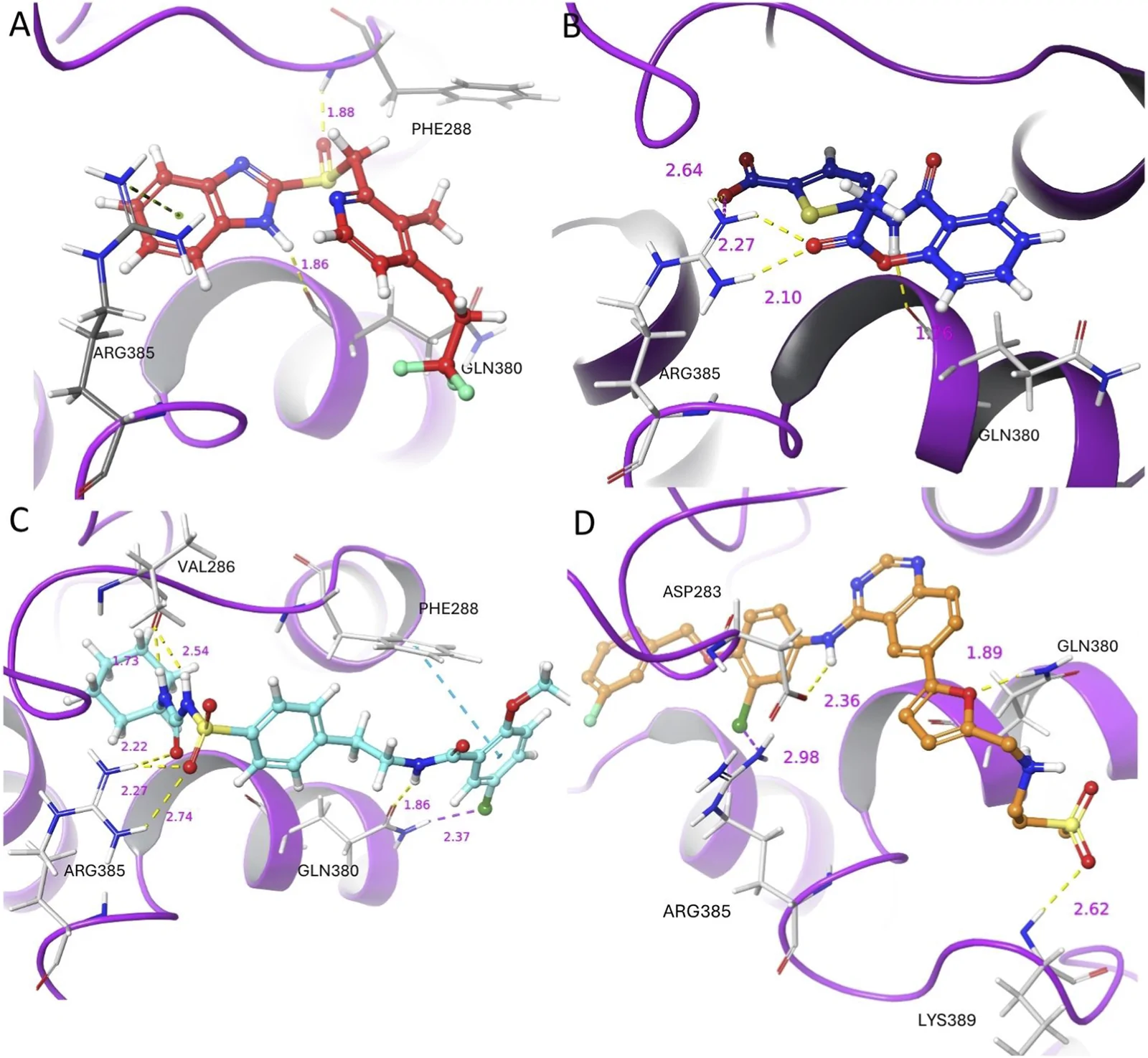
Binding pose of **(A)** Lansoprazole **(B)** Nitazoxanide **(C)** Glyburide and **(D)** Lapatinib into Site 1. The yellow dashes represent H-bond interactions; green dashes the π-cation contacts; cyan dashes the π–π interactions and pink dashes the salt bridge.

The results of the comprehensive computational analysis of the two identified putative binding sites suggested that the virtual screening campaign on the FDA library should target both binding sites to repurpose approved drugs as modulators of NR2F2 transcriptional activity modulators. First, we docked the FDA library by using XP docking and then calculated MM-GBSA free energy. The analysis identified six compounds as promising binders for Site 1 (Ticagrelor, Aliskiren, Lansoprazole, Lapatinib, Glyburide, and Nitazoxanide) and two compounds as promising binders for Site 2 (Pentostatin and Ribavirin). Ticagrelor is a P2Y12 platelet inhibitor used in patients with a history of myocardial infarction or with acute coronary syndrome (ACS) to prevent future myocardial infarction, stroke and cardiovascular death (Tao et al., 2022). Aliskiren is a direct renin inhibitor used to manage hypertension (Sureshkumar, 2008). Lansoprazole is a proton pump inhibitor used to help gastrointestinal ulcers heal, to treat symptoms of gastroesophageal reflux disease (GERD), to eradicate *Helicobacter pylori*, and to treat hypersecretory conditions such as Zollinger-Ellison Syndrome (Strand et al., 2017). Lapatinib is an antineoplastic agent and tyrosine kinase inhibitor used for the treatment of advanced or metastatic HER-positive breast cancer in patients who received prior chemotherapeutic treatments (Schlam and Swain, 2021). Glyburide is a sulfonylurea used in the treatment of non-insulin dependent diabetes mellitus (Gangji et al., 2007). Nitazoxanide is a thiazolidine anti-infective used to treat infections by protozoa, helminths, anaerobic bacteria, microaerophilic bacteria, and viruses (Rossignol, 2014). Pentostatin is for the treatment of hairy cell leukemia refractory to alpha interferon (Grever et al., 2003). Ribavirin is a guanosine nucleoside used to treat some forms of Hepatitis C (Thomas et al., 2012). At Site 1, despite the structural diversity of the identified compounds, Ticagrelor, Aliskiren, Lansoprazole, Lapatinib, Glyburide, and Nitazoxanide showed a conserved interaction pattern, with Arg385 emerging as the main residue. Additional contacts with Gln380 further contributed to the ligand–protein complex stabilization. Representative binding modes of four compounds are described below, whereas the complete interaction profiles of all identified ligands are summarized in Table 3. Lansoprazole established a hydrogen bond between its sulfonamide moiety and Phe288 (1.88 Å), as well as an additional hydrogen bond between the benzimidazole nitrogen and Gln380 (1.86 Å). Moreover, the aromatic ring of the benzimidazole scaffold was involved in a π–cation interaction with Arg385, contributing to ligand stabilization (Figure 13A). Nitazoxanide formed a hydrogen bond between the benzamide nitrogen and Gln380 (1.26 Å). In addition, the carbonyl oxygen of the 2-(acetyloxy)phenyl moiety established two hydrogen-bond interactions with Arg385 (2.10 and 2.27 Å), while the nitro group of the 5-nitro-2-thiazolyl ring established both salt-bridge and hydrogen-bond interactions with Arg385 (2.64 Å) (Figure 13B). The sulfonylurea moiety of Glyburide formed three hydrogen-bond interactions with Arg385 (2.22, 2.27, and 2.74 Å) through its oxygen atoms. The nitrogen atoms of the sulfonylurea region further engaged Val286 through two hydrogen bonds (1.73 and 2.54 Å). Additional contacts with Gln380, including a hydrogen bond with the NH group of the methoxybenzamide moiety (1.86 Å) and a halogen bond involving the chlorine atom (2.37 Å), further supported ligand binding. Moreover, the methoxybenzamide portion established a π–π interaction with Phe288, contributing to the stabilization of the ligand within the binding pocket (Figure 13C). As shown in Figure 13D, Lapatinib formed a hydrogen bond with Asp283 (2.36 Å) through the secondary amine bridging the quinazoline core and the chlorophenyl moiety. The furan ring contributed to ligand stabilization through a hydrogen bond with Gln380 (1.89 Å), while the chlorophenyl moiety established a halogen bond with Arg385 (2.98 Å). In addition, the sulfone group formed a hydrogen bond with Lys389 (2.62 Å), further stabilizing the ligand within the binding pocket.

**TABLE 3:** Target–ligands interactions observed in the docking analysis.

Site 1			
Cmpd	Residue	Int.*	Distance (Å)
Lanzoprazole	Phe288	HB	1.88
	**Gln380**	HB	1.86
	**Arg385**	π–cat	​
Nitazoxanide	**Gln380**	HB	1.26
	**Arg385**	HB	2.10
	**Arg385**	HB	2.27
	**Arg385**	HB	2.64
	**Arg385**	SB	​
Ticagrelor	Val286	HB	1.80
	Phe288	π– π	​
	**Gln380**	HB	2.14
	**Arg385**	HB	1.81
	**Arg385**	HB	2.80
Aliskiren	Glu379	HB	1.86
	**Gln380**	HB	2.21
	**Arg385**	HB	2.44
	Lys389	HB	1.79
Glyburide	Val286	HB	1.73
	Val286	HB	2.54
	Phe288	π– π	​
	**Gln380**	HB	1.86
	**Gln380**	HaB	2.37
	**Arg385**	HB	2.22
	**Arg385**	HB	2.27
	**Arg385**	HB	2.74
Lapatinib	Asp283	HB	2.36
	**Gln380**	HB	1.89
	**Arg385**	HaB	2.98
	Lys389	HB	2.62

Site 2			
Cmpd	Residue	Int.*	Distance (Å)
Pentostatin	Pro192	HB	1.97
	**Gln199**	HB	2.54
	**Arg218**	π–cat	​
	Ser222	HB	1.67
Ribavirin	Glu189	HB	2.09
	Arg195	HB	1.77
​	**Gln199**	HB	2.35
	**Arg218**	HB	2.35

Int.* is for interactions, HB is for H-bonds, HaB is Halogen-bond, π–cat. is π–cation, SB is Salt bridge, π–π is π–π stacking. Bold values indicate amino acid residues shared in common. “Res” refers to an amino acid residue.

At Site 2, Pentostatin and Ribavirin primarily interact with Arg218, with additional hydrogen-bond interactions involving Gln199. These conserved contacts define a stable binding mode within Site 2, while additional interactions with surrounding residues further contribute to complex stabilization. The complete interaction profiles of Pentostatin and Ribavirin are summarized in Table 3.

### 3.7 Experimental validation of *in silico*–identified NR2F2 modulators

**FIGURE 14:**
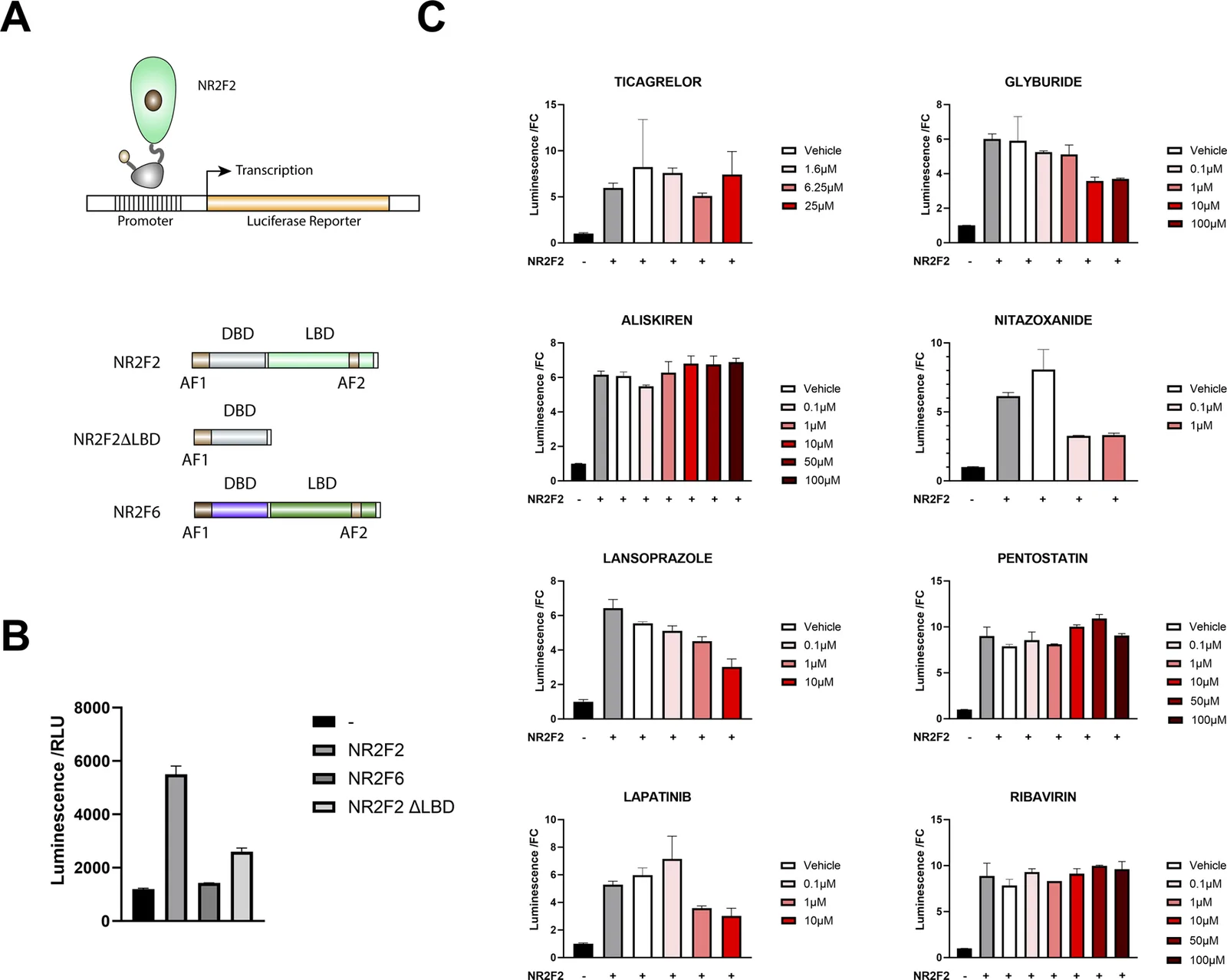
Hela cells are co-transfected with expression vectors for the indicated proteins (NR2F2, NR2F6, NR2F2ΔLBD), a NanoLuc reporter construct containing an NR2F2-specific response element upstream of a minimal promoter (pNL2.1_NR2F2) and a Firefly luciferase plasmid as an internal control (pGL4.54_Luc2/TK). **(A)** Schematic representation of the luciferase reporter system (upper panel) and domain organization of the overexpressed proteins (lower panel). LBD, ligand-binding domain; DBD, DNA-binding domain; AF, activation function (transcriptional activation domain). **(B)** 24 h post transfection, luciferase assay was carried out according to the Nano-Glo Dual-Luciferase Reporter Assay System guidelines (Promega). The graph is representative of two independent experiments. **(C)** Hela cells were seeded in 96 well plates in the presence of DMEM/F12 media supplemented with 10% FBS. After 24 h, cells were co-transfected with expression vectors for NR2F2 or an empty vector, as a control, pNL2.1_NR2F2 and pGL4.54_Luc2/TK. 18 h post-transfection, cells were treated with the depicted compounds for 24 h at the indicated concentrations. Cells were lysed according to the Nano-Glo Luciferase assay guidelines (Promega). Luminescence was measured in a GloMAx microplate reader and normalized to a co-transfected Firefly luciferase plasmid as an internal control.

To evaluate whether the identified compounds functionally interact with NR2F2, we assessed their ability to modulate NR2F2-dependent transcriptional activity. We generate an *ad hoc* reporter assay by cloning a NR2F2-specific response element, found in the NGF1A promoter (Wang et al., 2021), upstream of a minimal promoter for the NanoLuc luciferase protein (Promega) (Figure 14A). As shown in Figure 14B, NR2F2 overexpression selectively increases the reporter activity in Hela cells while the cognate ONR, NR2F6, exerts no activity. A deleted derivative of NR2F2 lacking the ligand binding domain (LBD) of the ONR is also devoid of any specific activity and provides further support to the specificity of the assay (Figure 14A). Indeed, the LBD of NR transcription factors is supposed to be responsible for their ligand binding. In the case of NR2F2, it has been demonstrated to account for the binding of 1-deoxysphingolipids, an endogenous natural ligand, able to modulate NR2F2 transcriptional activity (Wang et al., 2021). Among the tested compounds, Lapatinib, Glyburide, Nitazoxanide and Lansoprazole showed the ability to modulate NR2F2 dependent reporter activity. The observed dose response effect supports *in silico* findings (Figure 14C).

**FIGURE 15:**
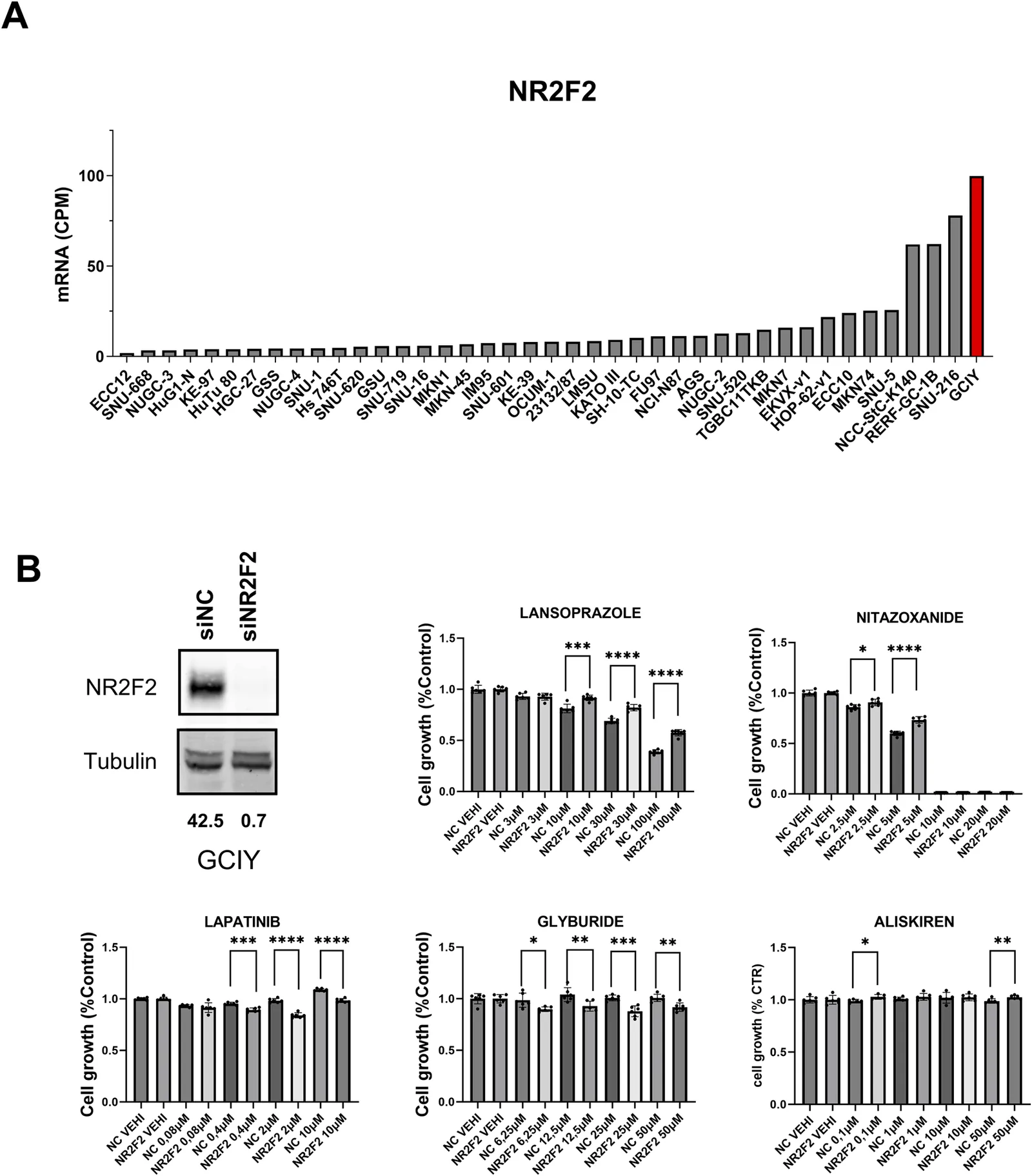
**(A)** The graph depicts NR2F2 expression levels (mRNA) across multiple gastric cancer cell lines according to the CCLE database (https://sites.broadinstitute.org/ccle). **(B)** GCIY gastric cancer cell line was transfected with siRNA targeting NR2F2 (siNR2F2) or a control sequence as a negative control (siNC). 48 h after transfection, cells were harvested and cell lysates subjected to Westernblot analysis (left panel) for NR2F2 and tubulin (loading control) detection. NR2F2 expression levels, assessed by densitometric analysis using Fiji (ImageJ), are indicated in arbitrary units (A.U.). In parallel silenced cells were cultured for further 72 h in the presence or absence of the indicated compounds, at the indicated concentration (x-axis), and cell growth was assessed via Sulphorodamine Assay (Voigt, 2005) or CellTiter-Glo® Luminescent Cell Viability Assay (Promega). The asterisks indicate the significan p-values calculated for the comparisons between each siNR2F2 and siNC point in the curves, using unpaired t-test (*p < 0.05; **p < 0.01; ***p < 0.001; ****p < 0.0001).

The identified compounds are currently approved by regulatory agencies for clinical use in cancer and other pathological conditions. In addition, preclinical studies have demonstrated their antitumor effects on gastric cancer cell proliferation (Cheng, 2024; Kao et al., 2023; Qian et al., 2008; Ribeiro et al., 2023). Accordingly, we assessed their impact on cell proliferation in the gastric cancer GCIY since transcriptome profiling revealed that GCIY were among the gastric cancer cell lines exhibiting the highest expression levels of NR2F2 (Figure 15A). In parallel, we silenced ONR using a pool of two validated siRNAs (Ambion™ Silencer™ Select siRNAs) and evaluated the response of the silenced cells to the previously identified compounds. As shown in Figure 15B (left, upper panel), NR2F2 was efficiently silenced in the selected gastric cancer cell line. In this cellular background, Lansoprazole and Nitazoxanide exert an antiproliferative effect that is mitigated by NR2F2 silencing (Figure 15). Indeed, dose–response analysis performed in the presence of NR2F2 siRNAs revealed a reduced ability of the compounds to inhibit cell growth across all effective concentrations tested (Figure 15B, right, upper panel). In contrast, Lapatinib and Glyburide did not significantly affect cell proliferation. Interestingly, under these conditions, siRNA treatment alone produced a modest reduction in cell growth, which was evident at all tested concentrations (excluding the lowest dose of Lapatinib) and observed only in the presence of the analyzed compounds (Figure 15B, bottom panel). To assess the specificity of the siRNA effects on gastric cancer cell growth, we performed the same experiments using Aliskiren, a compound predicted to bind NR2F2 but unable to affect NR2F2-dependent transcription (Figure 14). Aliskiren did not affect cell proliferation in the GCIY cell line, and NR2F2 silencing did not consistently alter cell growth either in the presence or absence of the compound.

Overall, the data described above suggest that bound NR2F2 could affect cancer cell growth, sensitizing cells to anti-proliferative effects.

## 4 Discussion

NR2F2 (COUP-TFII) is a ligand-competent orphan nuclear receptor with pivotal roles in embryonic development, tissue homeostasis, and cancer, where it functions as a context-dependent transcriptional regulator of lineage and metabolic programs (Sajinovic and Baier, 2023). Although long regarded as ligand-independent, structural and reporter studies have indicated that the NR2F2 ligand-binding domain (LBD) retains a functional pocket capable of accommodating small molecules, and that LBD engagement can reshape receptor-mediated transcription (Kruse et al., 2008). These studies have proposed that unligated NR2F2 falls in an autorepressed form by preventing coactivators recruitment, ligand binding should release autorepression, allowing NR2F2 transcriptional activity. In particular, Wang et al. identified 1-deoxysphingolipids as endogenous NR2F2 ligands, demonstrating direct binding to the NR2F2 LBD. Together, these findings support the emerging view that NR2F2 activity can be tuned by small molecules. In detail, the present study aimed to evaluate the potential druggability of the ligand-binding domain of NR2F2 related to the only trascriptional inhibitor comprehensively characterized by experimental evidence (CIA1) (Wang et al., 2020). To this aim, the ligand binding domain was mapped to identify potential binding sites, designated as Site 1 and Site 2. Molecular docking was employed to elucidate the binding mode and interactions between CIA1, the known inhibitor, and the two active sites. Subsequently, the dynamic landscape of the docking complexes was evaluated using molecular dynamics, binding pose metadynamics, and MM-GBSA studies. These analyses revealed the druggability of both sites. Specifically, in Site 1 the H-bond interaction with Gln380 observed in docking studies was lost due to CIA1 shifting from its original docking position and establishing new interactions, such as H-bond and hydrophobic interactions with Ile209 and other residues, Glu210, Ala267 and Arg385. On the other hand, in Site 2, the H-bond with Arg218 was maintained throughout the molecular dynamics replicas and reinforced by other interactions, particularly with Ser222. Binding pose metadynamics and free energy calculation confirmed the trend observed in the first analysis. This comprehensive computational characterization of the ligand binding domain of NR2F2 elucidates the crucial role of these residues in ligand binding in the two active sites. The virtual screening campaign performed on the FDA-approved drug library identified lapatinib, glyburide, nitazoxanide, and lansoprazole as promising NR2F2 modulators. Although these compounds differ in their chemical structures, they showed a common interaction pattern within the predicted binding sites, particularly involving Arg385, suggesting a possible role of this residue in ligand recognition and NR2F2 modulation. The docking analysis helped to define the binding modes of these compounds and highlighted key protein–ligand interactions that may be considered for the further optimization of NR2F2 modulators. In particular, these interactions may guide future scaffold optimization and scaffold hopping strategies aimed at preserving the molecular features required for NR2F2 activity modulation. In addition, bioisosteric modifications could be investigated to improve ligand affinity and selectivity while maintaining relevant interactions within the binding sites.

To test whether our candidate compounds functionally engage NR2F2, we established a NanoLuc reporter driven by an NR2F2-specific response element derived from the NGF1A promoter, previously mapped as a COUP-TF responsive region (Wang et al., 2020). In HeLa cells, NR2F2 overexpression robustly increased reporter activity, whereas the cognate orphan receptor NR2F6 had no effect, indicating that this element operates as a selective NR2F2 readout in our experimental context. Importantly, an NR2F2 derivative lacking the LBD failed to activate the reporter, demonstrating that (i) transactivation in this system requires intact LBD-dependent regulatory surfaces, and (ii) the assay is poised to detect ligand-mediated modulation rather than reflecting only DNA occupancy. This observation aligns with canonical nuclear-receptor mechanisms, where the LBD governs ligand recognition and co-regulator exchange, and specifically with NR2F2 biology, in which the LBD mediates binding of 1-deoxysphingolipids that modulate transcription at physiological concentrations.

Using this validated platform, we identified lapatinib, glyburide, nitazoxanide, and lansoprazole as modulators of NR2F2-dependent reporter activity. The presence of reproducible dose–response relationships argues against nonspecific effects. Interestingly, previous studies have reported that these compounds can influence selected nuclear receptor pathways. Lapatinib functionally modulates nuclear-receptor pathways—such as ERRα, ERα and AR—through inhibition of EGFR/HER2 and downstream signaling (Deblois et al., 2016; Shiota et al., 2015; Wang et al., 2011). Glyburide was reported as a direct PPARγ ligand and modulator of its signalling (Fukuen et al., 2005; Haas et al., 2024). Glyburide also modulates FXR- and PRR–reporter dependent transcription (Steri et al., 2010; Van Giersbergen et al., 2002). Nitazoxanide was shown to functionally modulate PPARγ signalling (Elaidy et al., 2018). Lansoprazole acts as a functional agonist of LXRα/LXRβ and PXR (Cronican et al., 2010; Novotna and Dvorak, 2014). For Lapatinib, Glyburide, Nitazoxanide and Lansoprazole, direct links to NR2F2 have not, to our knowledge, been reported. Lapatinib is an orally active small-molecule tyrosine kinase inhibitor that targets the epidermal growth factor receptor (EGFR/HER1) and human epidermal growth factor receptor 2 (HER2/ErbB2). It is primarily used in the treatment of HER2-positive breast cancer and other malignancy characterized by HER2 amplification such as gastric cancer (Cheng, 2024; Schlam and Swain, 2021). Glyburide (also known as glibenclamide) is a second-generation sulfonylurea widely used for the treatment of type 2 diabetes mellitus. It lowers blood glucose levels by stimulating insulin secretion through inhibition of ATP-sensitive potassium (K_ATP) channels in pancreatic β-cells and has additionally been reported to exhibit anti-inflammatory and ion-channel–modulating effects (Gangji et al., 2007). Glyburide has been reported to inhibit the proliferation of the gastric cancer cell line MGC-803 (Qian et al., 2008). Nitazoxanide is a broad-spectrum antiparasitic agent approved for the treatment of protozoal infections such as cryptosporidiosis and giardiasis. Beyond its antiparasitic activity, nitazoxanide has demonstrated antiviral and immunomodulatory properties, largely through interference with host-regulated metabolic and signaling pathways required for pathogen replication (Rossignol, 2014). Interestingly, Nitazoxanide has been used for the treatment of *Helicobacter pylori* and has shown antiproliferative activity in gastric cancer cell lines (Lee et al., 2020; Ribeiro et al., 2023). Lansoprazole is a proton pump inhibitor (PPI) commonly prescribed for acid-related gastrointestinal disorders, including gastroesophageal reflux disease and peptic ulcer disease (Strand et al., 2017). It acts by irreversibly inhibiting the gastric H^+/^K^+^-ATPase proton pump, thereby suppressing gastric acid secretion. Interestingly, also Lansoprazole has shown to exert an antiproliferative activity on gastric cancer cell lines (Kao et al., 2023). All the identified compounds had previously been reported to exert antiproliferative effects in gastric cancer cell lines; a malignancy for which therapeutic options remain limited. To further validate the functional interaction between these compounds and NR2F2, we silenced NR2F2 expression in the GCIY gastric cancer cell line, which exhibited the highest endogenous expression of the receptor among the tested models. Under our experimental conditions, only lansoprazole and nitazoxanide consistently retained antiproliferative activity. Notably, NR2F2 silencing significantly impaired the growth-inhibitory effects of both compounds, suggesting that their antiproliferative activity is at least partially mediated through NR2F2-dependent mechanisms.

## 5 Conclusion

Taken together, our data extend current evidence that NR2F2 is a ligandable orphan receptor whose transcriptional output can be pharmacologically tuned. The convergence between *in silico* predictions and LBD-dependent reporter modulation supports the interpretation that lapatinib, glyburide, nitazoxanide, and lansoprazole represent functional interactors of NR2F2. Future investigations using dedicated biophysical binding assays (e.g. SPR) will be required to confirm whether these compounds directly bind NR2F2 and to determine their binding affinity, specificity, and interaction kinetics. Importantly, lansoprazole and nitazoxanide were demonstrated to exert antiproliferative activity via NR2F2. Overall, these findings provide new chemical tools for probing NR2F2 biology and nominate clinically used molecules as starting points for therapeutic or repurposing strategies targeting NR2F2-dependent programs. Building on these findings, future efforts, integrating structure-guided optimization of the identified scaffolds with further exploration of clinically approved compounds may facilitate the development of NR2F2-targeted drug repurposing strategies.

## Data Availability

The raw data supporting the conclusions of this article will be made available by the authors, without undue reservation.
